# m^6^A‐Modified SNRPA Controls Alternative Splicing of ERCC1 Exon 8 to Induce Cisplatin Resistance in Lung Adenocarcinoma

**DOI:** 10.1002/advs.202404609

**Published:** 2024-11-18

**Authors:** Weina Fan, Jian Huang, Fanglin Tian, Xin Hong, Kexin Zhu, Yuning Zhan, Xin Li, Xiangyu Wang, Xin Wang, Li Cai, Ying Xing

**Affiliations:** ^1^ The Fourth Department of Medical Oncology Harbin Medical University Cancer Hospital 150 Haping Road Harbin 150081 China; ^2^ NHC and CAMS Key Laboratory of Molecular Probe and Targeted Theranostics Harbin Medical University Harbin 150001 China

**Keywords:** alternative splicing, cisplatin resistance, DNA repair, ELAVL1, ERCC1, SNRPA

## Abstract

Alternative splicing (AS) generates protein diversity and is exploited by cancer cells to drive tumor progression and resistance to many cancer therapies, including chemotherapy. SNRPA is first identified as a spliceosome‐related gene that potentially modulates resistance to platinum chemotherapy. Both the knockout or the knockdown of SNRPA via CRISPR/Cas9 and shRNA techniques can reverse the resistance of cisplatin‐resistant lung adenocarcinoma (LUAD) cells to cisplatin. SNRPA overexpression enhanced the resistance of cisplatin‐sensitive LUAD cells. Gene Ontology (GO) analysis reveals that SNRPA is associated with DNA damage repair. Depletion of SNRPA induced ERCC1 exon 8 skipping and reduced ERCC1–XPF complex formation, whereas SNRPA overexpression exerted the opposite effect. siRNAs targeting isoforms containing ERCC1 exon 8 [ERCC1‐E8 (+)] reversed SNRPA‐enhanced cisplatin resistance and DNA damage repair. Furthermore, the IGF2BP protein, an m^6^A reader, and the ELAVL1 protein, an RNA stabilizer recruited by IGF2BP1, are found to bind to the SNRPA mRNA. ELAVL1 promoted cisplatin resistance, DNA repair and ERCC1‐E8 (+) expression in an SNRPA‐dependent manner. In a mouse xenograft model, SNRPA‐*KO* CRISPR enhanced the sensitivity of LUAD cells to cisplatin. Overall, this study illuminates the role of SNRPA in platinum‐based drug resistance, thereby providing a novel avenue to potentially enhance chemosensitivity and improve the prognosis of patients with LUAD.

## Graphical abstract

ELAVL1, in conjunction with IGF2BP, promotes the recognition of m^6^A methylation, thereby increasing SNRPA mRNA stability and expression. This elevated SNRPA expression subsequently promotes the formation of ERCC1‐E8(+), which includes exon 8 and encodes the XPF protein‐binding domain. The resulting increase in the production of the ERCC1‐XPF complex enhances DNA repair, leading to enhanced cisplatin resistance.

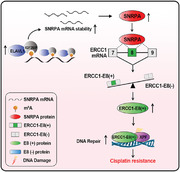

## 1 Introduction

Lung adenocarcinoma (LUAD) is one of the most lethal cancers and is characterized by difficulty in early diagnosis, high rates of recurrence and metastasis, and a dismal prognosis.^[^ ^1^, ^2^ ^]^ Depending on the stage, treatments for LUAD vary and include surgery, chemotherapy, radiotherapy, targeted therapy, and immunotherapy.^[^ ^3^ ^]^ The majority of LUAD cases are diagnosed at an advanced stage and are inoperable, with limited therapeutic alternatives; thus, chemotherapy remains the front‐line strategy for treating LUAD.^[^ ^4^ ^]^ Platinum compounds, including cisplatin [also known as cis‐diamminedichloroplatinum (II), CDDP], remain the cornerstone of LUAD chemotherapy.^[^ ^5^ ^]^ Through DNA–platinum adduct formation and the stimulation of apoptotic signaling in cancer cells, cisplatin therapy provides patients a remarkable survival advantage; however, cisplatin resistance restricts its therapeutic effectiveness and contributes to cancer recurrence in patients with LUAD.^[^ ^6^, ^7^ ^]^ Therefore, the discovery of reliable prognostic indicators for the response or resistance to cisplatin and a deeper understanding of the molecular mechanisms underlying cisplatin chemoresistance are essential.

Apart from intrinsic causes, cisplatin resistance in LUAD can be acquired through various mechanisms, which can be categorized as follows: increased DNA repair or tolerance, amplified cisplatin detoxification, inhibition of apoptosis, and reduced intracellular accumulation of cisplatin.^[^ ^8^, ^9^ ^]^ Cisplatin primarily targets genomic DNA, resulting in a multitude of DNA lesions that impede transcription and replication.^[^ ^10^ ^]^ To fix the DNA damage caused by cisplatin, cells activate the DNA damage response (DDR) and engage an intricate network of mechanisms, including DNA damage repair pathways.^[^ ^9^ ^]^ In actuality, cisplatin‐induced DNA damage is repaired via nucleotide excision repair (NER), interstrand crosslink (ICL) repair, mismatch repair (MMR), homologous recombination (HR) and nonhomologous end joining (NHEJ) in a variety of cancers, including lung cancer.^[^ ^11^ ^]^ Our previous research revealed that TRIM44 enhances BRCA1‐mediated homologous recombination, thereby facilitating cisplatin chemoresistance through the deubiquitylation of FLNA.^[^ ^12^ ^]^

The structure‐specific endonuclease ERCC1‐XPF, a multifunctional heterodimer, is essential for DNA damage repair, including NER, HR, and ICL repair.^[^ ^13^, ^14^ ^]^ The essential interaction between the ERCC1 and XPF proteins involves the dimerization of their hydrophobic C‐terminal regions through double helix–hairpin–helix motifs within their HhH2 domains, spanning a distance of 1534 Å, which results in stable heterodimer formation.^[^ ^15^ ^]^ This dimerization serves to protect each protein from degradation due to aggregation.^[^ ^16^ ^]^ Utilizing cross‐saturation techniques, ERCC1 residues from Arg234 to Leu294 have been shown to interact with XPF residues ranging from Gln849 to Ala906.^[^ ^16^ ^]^ Elevated expression of ERCC1 is associated with poor therapeutic outcomes following cisplatin administration in multiple malignancies, including LUAD.^[^ ^17^, ^18^ ^]^ By inhibiting the protein–protein interaction between ERCC1 and XPF, this inhibitor enhances the sensitivity of LUAD cells to platinum‐based drugs.^[^ ^19^ ^]^ However, the specific factors and molecular mechanisms that govern the interaction between ERCC1 and XPF remain to be fully elucidated.

Alternative splicing (AS) occurs in over 95% of human protein‐encoding genes and serves as a major evolutionary driver as well as the primary cause of proteome diversity.^[^ ^20^ ^]^ Dysregulated RNA splicing is a prevalent factor contributing to genetic diseases, including cancer.^[^ ^21^ ^]^ Cancer‐associated splicing dysregulation increases cell proliferation, inhibits apoptosis, promotes the metastatic potential, enables immunological evasion, and confers drug resistance.^[^ ^22^, ^23^ ^]^ Eukaryotic precursor mRNA (pre‐mRNA) splicing is orchestrated by a large and intricate ribonucleoprotein complex known as the spliceosome.^[^ ^24^ ^]^ Its roles include defining splice locations and catalyzing RNA splicing reactions.^[^ ^25^ ^]^ The spliceosome comprises five small nuclear ribonucleoproteins (snRNPs): U1, U2, U4, U5, and U6. Each snRNP consists of a uridine‐rich small nuclear RNA (U snRNA) that ranges from 100 to 300 nucleotides in length, Sm proteins (including SNRPB/B′, D1, D2, D3, E, F, and G), and numerous associated proteins.^[^ ^26^ ^]^ Key spliceosome‐related genes have been identified and experimentally validated in the context of malignant tumors.^[^ ^27^, ^28^, ^29^ ^]^ As an illustration, SNRPB is known to modulate cisplatin resistance in lung cancer via the ERK signaling pathway.^[^ ^30^ ^]^ However, the specific spliceosome‐associated genes related to cisplatin resistance are still largely unidentified.

In this study, SNRPA was identified as a novel gene associated with platinum resistance using computational techniques. We established stable cell lines with SNRPA knockout, knockdown, and overexpression to confirm the role of SNRPA in cisplatin chemoresistance. The phenotypes of the cisplatin‐resistant LUAD cells were found to be modulated by SNRPA expression. We discovered that SNRPA increases the production of ERCC1 transcripts that include exon 8 [referred to as ERCC1‐E8(+) or simply E8 (+)] and that SNRPA‐induced cisplatin resistance, as well as DNA damage repair, were partially dependent on ERCC1‐E8(+). Furthermore, the stability of the SNRPA mRNA was shown to be epigenetically regulated by embryonic lethal abnormal vision‐like protein 1 (ELAVL1), which affected SNRPA expression. These findings increase our understanding of the functions, mechanisms, and epigenetic regulation of SNRPA, thus providing potential therapeutic targets for combating chemoresistance.

## 2 Results

### 2.1 Identification of Spliceosome‐Related Genes Governing Platinum‐Based Drug Resistance

**Figure 1:**
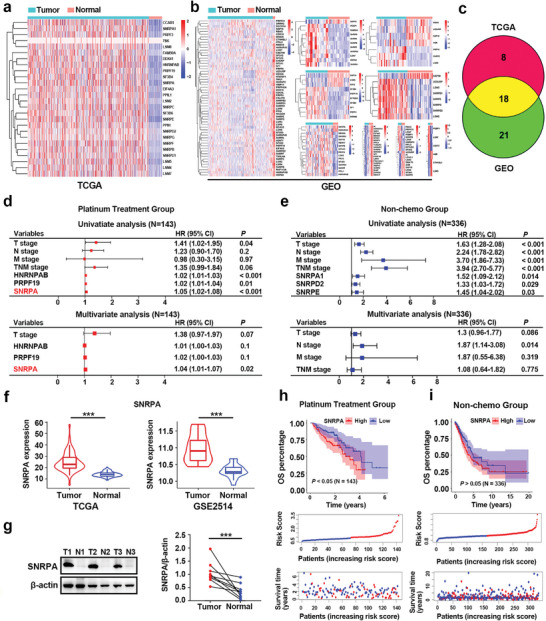
Identification of spliceosome‐related genes that modulate platinum‐based drug resistance. a,b) Heatmaps showcasing the differential gene expression profiles, highlighting genes that are dysregulated in LUAD samples relative to healthy lung tissue counterparts based on TCGA (a) and GEO (b) databases. c) Venn diagram depicting the intersection of upregulated spliceosome‐related genes obtained from TCGA and GEO databases. d,e) Forest plots of overall survival (OS) revealed the results of univariate and multivariate regression analyses of the platinum‐based treatment group (d) and the non‐chemo group (e). f) Violin plots depicting the comparative analysis of SNRPA mRNA expression profiles, contrasting LUAD tissues with non‐neoplastic lung counterparts, based on the TCGA and GEO databases. g) Western blot revealed the protein expression of SNRPA using fresh frozen LUAD (n = 9) and paired normal adjacent tissue samples (n = 9), and the other results are shown in Figure S2a (Supporting Information). h,i) Kaplan–Meier survival plots delineating the OS stratification for patients with LUAD who were categorized on the basis of elevated versus diminished SNRPA expression levels in the platinum treatment group (h) and non‐chemo group (i) (Log rank test; TCGA: n = 592; GSE2514: n = 39). All data are presented as the mean ± SD (n = 3). The *p* values in panels (f) and (g) were calculated by Student's t‐test. ***p < 0.001.

To identify spliceosome‐related genes potentially involved in chemoresistance to platinum‐based drugs, we obtained the annotation information for 137 spliceosome‐related genes from the Spliceosome database (http://spliceosomedb.ucsc.edu/) (Table S1, Supporting Information). This study included 1205 patients whose gene expression information and clinicopathological features were available in The Cancer Genome Atlas (TCGA, n = 518) and the Gene Expression Omnibus (GEO, n = 687) databases. A flowchart of the bioinformatics analysis is detailed in Figure S1 (Supporting Information). Compared with those in normal lung tissues, the expression of spliceosome‐related genes that were differentially expressed in LUAD tissues was determined by analyzing transcriptome gene expression datasets from lung tissues in TCGA and GEO databases (**Figure** **1**a,b). A Venn diagram revealed 18 dysregulated spliceosome‐related genes at the intersection of TCGA and GEO datasets (Figure 1c). Within TCGA‐LUAD cohort, for 143 patients who received platinum‐based chemotherapy (platinum treatment group) and 348 who received no chemotherapy (non‐chemo group), we conducted a Cox regression analysis. Univariate and multivariate analyses identified elevated SNRPA expression as an independent prognostic indicator for OS in the cohort treated with platinum‐based chemotherapy but not in the non‐chemo group (Figure 1d,e). These results prompted us to choose SNRPA as a candidate spliceosome‐related gene for further investigation into its role in cisplatin resistance.

Compared to normal lung tissues, hierarchical clustering analysis revealed that SNRPA expression was elevated in LUAD tissues, based on the public datasets (Figure 1f). Western blot further confirmed increased expression of the SNRPA protein in fresh frozen LUAD tissue samples (Figure 1g; Figure S2a, Supporting Information). The Kaplan–Meier analysis indicated that high SNRPA expression predicted a shorter overall survival time for LUAD patients who received platinum‐based chemotherapy; however, no significant difference was observed in the non‐chemo group (Figure 1h,i).

### 2.2 SNRPA Reinforces LUAD Cell Cisplatin Resistance both In Vitro and In Vivo

**Figure 2:**
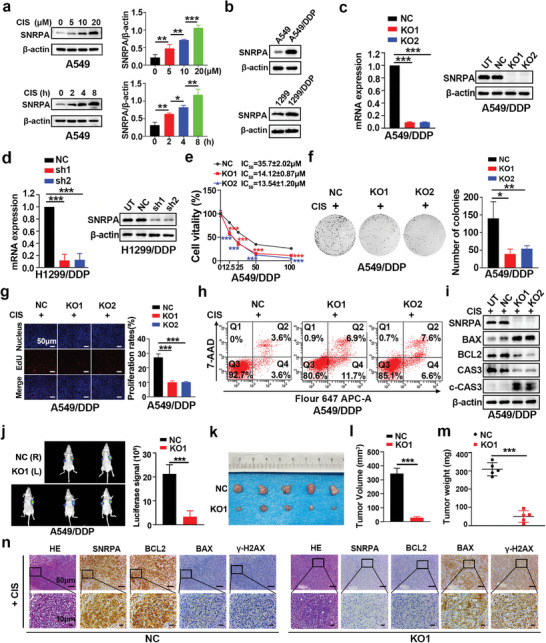
The depletion of SNRPA reverses the cisplatin chemoresistance of cisplatin‐resistant cells. a) Western blot representing the levels of SNRPA expression after treatment with increasing cisplatin dosages or the time increasing durations of cisplatin treatment (left panel). The bar graphs showed SNRPA protein expression based on the average grayscale value of the target protein (right panel). b) Western blot analyses demonstrating the quantification of SNRPA protein expression in cell lines characterized by sensitivity and resistance to cisplatin treatment. c) SNRPA expression in A549/DDP cells transfected with CRISPR negative control clones (NC), the SNRPA CRISPR/Cas9 knockout plasmid‐1 (KO‐1) or the SNRPA CRISPR/Cas9 knockout plasmid‐1 (KO‐2). d) Comparative analysis of both the mRNA and protein expression levels of SNRPA in H1299/DDP cells transfected with shNC (NC), shSNRPA‐1 (sh‐1), or shSNRPA‐2 (sh‐2). e) CCK‐8 analysis, after 48 h of cisplatin treatment, illustrated cell viability across the designated cellular groups (n = 4). f) Colonies were visualized by staining with crystal violet (left panel), while the bar graphs provided a statistical evaluation of the colony counts (right panel). g) EdU assays of the proliferation of the indicated cells in a 10 µM cisplatin solution. h) Representative images illustrating the results of Annexin V‐Fluor 647 APC/7‐AAD dual staining to evaluate apoptosis in cells treated with 10 µM cisplatin for 24 h. i) The expression of apoptosis‐associated proteins in A549/DDP cells. j) The left panel displayed representative bioluminescence images of xenograft tumors generated from NC or KO cells. Bar graphs were generated to quantify the bioluminescence luciferase signal emitted from the xenograft tumors, providing a statistical comparison between groups. k,m) The left panel showed images of xenograft tumors in the designated groups (k). These panels depict the outcomes of t‐tests applied to compare the mean tumor volume (l) and weight (m) across different groups. n) IHC analyses were performed on xenograft tumor tissues to examine the expression patterns of SNRPA, Bcl‐2, BAX, and *γ*‐H2AX. All data are presented as the mean ± SD (n≥3). The *p* values in panels (a), (c), (d), (f) and (g) were calculated using one‐way ANOVA. The *p* values in panels (j), (l) and (m) were calculated by Student's t‐test. The *p* values in panels (e) were calculated using two‐way ANOVA. *p < 0.05; **p < 0.01; ***p < 0.001.

To examine whether SNRPA contributed to cisplatin‐induced acquired drug resistance, we assessed the expression levels of SNRPA in cisplatin‐treated LUAD cells. We observed a gradual increase in SNRPA expression after treatment with increasing cisplatin doses or a prolonged treatment duration in the cisplatin‐sensitive A549 and H1299 cells, indicating a dose‐ and time‐dependent relationship (**Figure** **2**a; Figure S2b, Supporting Information). Furthermore, compared with their cisplatin‐sensitive parental counterparts, cisplatin‐resistant cells (A549/DDP and H1299/DDP) presented substantially increased SNRPA expression (Figure 2b; Figure S3a, Supporting Information).

Consequently, in functional studies, cisplatin‐resistant cells were used to establish a loss‐of‐function model, whereas parental LUAD cells served to create a gain‐of‐function model. Using CRISPR/Cas9 technology, we achieved successful knockout of the SNRPA mRNA and protein levels in SNRPA‐deficient CRISPR clones (KO1 and KO2) of A549/DDP via lentiviral constructs, as evidenced by comparisons with CRISPR negative control clones (NC) (Figure 2c). To enhance the validity of our findings, we designed specific shRNAs targeting SNRPA, as well as the negative control, and transfected them in H1299/DDP cells (Figure 2d). Notably, in the absence of cisplatin treatment, neither SNRPA knockdown nor knockout influenced the proliferation, apoptosis or DNA damage of chemotherapy‐resistant LUAD cells (Figure S2c‐e, Supporting Information). Similarly, in vivo experiments without cisplatin treatment revealed that SNRPA knockout did not affect tumor growth (Figure S2f‐i, Supporting Information).

Consistent with our expectations, SNRPA knockout cells demonstrated a lower 50% inhibitory concentration (IC_50_) when treated with progressively increasing concentrations of cisplatin (Figure 2e). Plate colony formation and EdU incorporation assays confirmed that the downregulation of SNRPA reduced cisplatin resistance (Figure 2f,g). Consistent with these findings, the resistance of H1299/DDP cells to cisplatin was reversed, as confirmed by CCK8, plate colony formation and EdU incorporation assays (Figure S3b‐d, Supporting Information). Flow cytometry analysis and Western blot were subsequently performed to evaluate the effect of SNRPA depletion on cisplatin‐induced apoptosis. SNRPA silencing noticeably escalated the proportion of apoptotic A549/DDP cells treated with cisplatin, which coincided with decreased expression of the anti‐apoptotic protein Bcl‐2 and an upsurge in the pro‐apoptotic protein BAX (Figure 2h,i; Figure S3e, Supporting Information). In the in vivo cisplatin resistance experiment, xenografts (NC and KO) derived from A549/DDP derivative cells (CRISPR negative control clones and SNRPA‐deficient CRISPR clones) were established in nude athymic mice. The experimental procedure for cisplatin injection was carried out as detailed in Figure S3g (Supporting Information). Compared with the NC group injected with NC cells, SNRPA knockout attenuated cisplatin resistance in LUAD cells located in the axillary region of nude mice, as evidenced by reduced luciferase activity (Figure 2j), decreased tumor volume (Figure 2k,l), and reduced tumor weight (Figure 2m). In consecutive tissue sections derived from xenografts, IHC staining demonstrated diminished BCL2 expression and augmented BAX expression in specimens from the KO group compared with those from the NC group (Figure 2n).

As indicated in Figures 2b and S3a (Supporting Information), cisplatin‐sensitive parental cells were employed to establish a gain‐of‐function model. We successfully established stable SNRPA‐overexpressing clones through lentiviral infection, which was validated by quantitative real‐time polymerase chain reaction (qRT‒PCR) and Western blot analyses (Figure S4a,b, Supporting Information). CCK‐8, colony formation, and EdU assays were employed to assess the cytotoxicity of cisplatin. The CCK‐8 results revealed that SNRPA overexpression induced the resistance of A549 and H1299 cells to cisplatin (Figure S5a, Supporting Information). Cell proliferation was evaluated through colony formation assays (Figure S5b, Supporting Information) and EdU experiments (Figure S5c, Supporting Information) under cisplatin treatment conditions. Flow cytometry and Western blot analysis elucidated that the overexpression of SNRPA inhibited cisplatin‐induced apoptosis (Figure S5d,e, Supporting Information). Consistent with the in vitro results, the spontaneous xenograft tumors formed by stable SNRPA‐overexpressing clones (SNRPA) displayed higher luciferase activity, as well as larger tumor volumes and weights, than those from the control (Ctrl) group (Figure S5f‐i, Supporting Information). However, without cisplatin treatment, overexpression of SNRPA did not affect in vitro LUAD cell proliferation, apoptosis or DNA damage and in vivo tumor growth (Figure S4c‐i, Supporting Information). Based on these findings, we concluded that SNRPA can induce cisplatin chemoresistance in LUAD.

### 2.3 SNRPA can Blunt Cisplatin‐Induced DNA Damage

**Figure 3:**
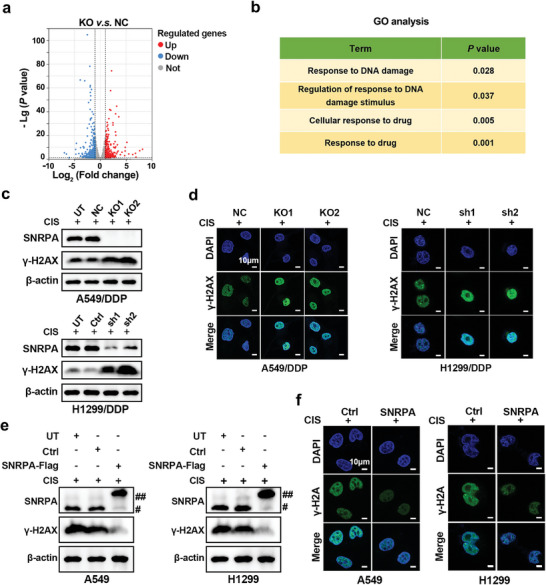
SNRPA promotes DNA damage repair. a) Volcano plot showing differentially expressed genes between SNRPA knockout cells (SNRPA‐KO1) and negative control cells (NC). b) Gene Ontology (GO) enrichment analysis was performed to delineate the biological processes involving SNRPA. The table illustrates that four pathways are significantly and statistically involved in the response to drug and response to DNA damage stimulus following SNRPA knockout. c) The expression levels of the *γ*‐H2AX protein in the specified cell lines following treatment with cisplatin and subsequent knockout or knockdown of SNRPA were assessed. ## represents exogenous Flag‐SNRPA, # represents endogenous SNRPA. d) Immunofluorescence co‐localization of *γ*‐H2AX and DAPI exhibited *γ*‐H2AX nuclear foci. The increased presence of *γ*H2AX indicated reduced DNA repair after DNA damage. e) The expression of the *γ*‐H2AX protein was assessed in the specified cells treated with cisplatin after SNRPA overexpression. f) Localization of *γ*‐H2AX and DAPI revealed *γ*‐H2AX nuclear foci. The decreased presence of *γ*H2AX indicated enhanced DNA repair after DNA damage.

To elucidate the mechanism behind SNRPA‐induced cisplatin resistance, we sequenced the mRNA transcriptomes of the NC and KO1 clones to perform a differential expression (DE) analysis. The DE analysis identified 324 upregulated genes and 368 downregulated genes after SNRPA knockout (*P* < 0.05, absolute log_2_ Fold change (FC) > 1) (**Figure** **3**a). Enrichment analysis of the DE genes using GO revealed many significant signal transduction pathways after SNRPA knockout. Among them, four pathways are significantly and statistically involved in the response to drug and response to DNA damage stimulus (Figure 3b). Phosphorylated H2AX (*γ*‐H2AX) is a known indicator of DNA damage and repair.^[^ ^31^, ^32^ ^]^ *γ*‐H2AX nuclear foci were observed to investigate the effects of SNRPA on DNA damage. The increased presence of *γ*‐H2AX foci following SNRPA knockout indicated a reduced DNA repair capability, as indicated by immunofluorescence staining and Western blot analyses (Figure 3c,d). In contrast, SNRPA overexpression enhanced DNA repair, contributing to a reduced presence of *γ*‐H2AX foci (Figure 3e,f). Animal experiments revealed an increase in *γ*‐H2AX expression in tumor tissues isolated from KO mice compared with those from the NC group (Figure 2n). These findings indicate that SNRPA enhances the repair of DNA damage induced by cisplatin in LUAD.

### 2.4 SNRPA can Control exon 8 Skipping of ERCC1 Gene and the Formation of the ERCC1–XPF Complex

**Figure 4:**
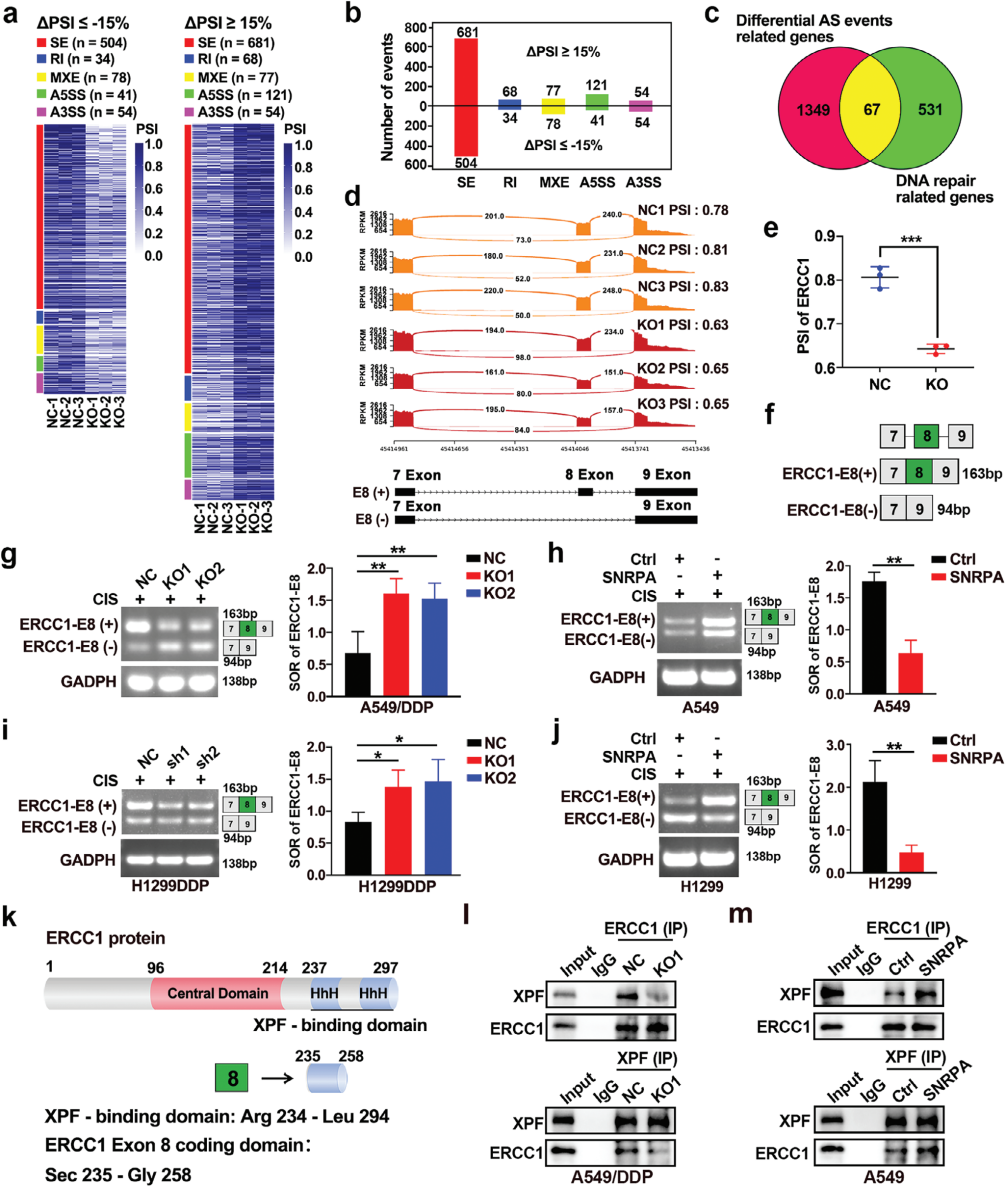
SNRPA suppresses exon 8 skipping of ERCC1 gene and promotes the formation of the ERCC1–XPF complex. a) Heatmap of significantly different AS events after SNRPA knockout (absolute ΔPSI ≥ 15%, *P* < 0.05). The color gradient indicates the percent spliced in (PSI) value of each event in each duplicate sample. b) Bar plot representing significantly different AS events. PSI = splice in/(splice in + splice out). ΔPSI = PSI(KO)‐PSI(NC). n = the number of AS events. Exon skipping (SE), intron retention (RI), mutually exclusive exons (MXE), alternative 5′ splice sites (A5SS), and alternative 3′ splice sites (A3SS). c) Venn diagram showing the intersection of 67 genes between differential AS event‐related genes and DNA repair‐related genes. d) Sashimi plot visualizing RNA‐seq reads mapped to exon 7, 8 and 9 of the ERCC1 mRNA after SNRPA knockout in A549/DDP cells. e) Scatter plot of the PSI for the ERCC1 exon 8 skipping event in NC cells and KO cells. f) Schematic representation (constitutive exon 8 and variable exons 7 and 9) of the splice variants [ERCC1‐E8(+) and ERCC1‐E8(−)]. g,i) Expression of ERCC1 transcripts with [ERCC1‐E8(+)] and without [ERCC1‐E8(−)] exon 8 after SNRPB knockdown in A549/DDP and 1299/DDP cells and the spliced out ratio [SOR, i.e., ERCC1‐E8(−)/ERCC1‐E8(+)]. h,j) Expression of ERCC1 transcripts with and without exon 8 after SNRPB overexpression in A549 and H1299 cells. Cropped blots are shown for the indicated ERCC1 isoforms or GAPDH. For uncropped blots, see Supporting Information (h‐j). k) Schematic diagram of the wild‐type long ERCC1 protein. The ERCC1 residues Arg234 to Leu294 constitute the XPF binding domain. Exon 8 of ERCC1 encodes Sec235 to Gly258. l,m) Immunoprecipitation assays were conducted to assess XPF, which was immunoprecipitated by ERCC1 after SNRPA dysregulation. All data are presented as the mean ± SD (n = 3). The *p* values in panels (e), (h) and (j) were calculated by Student's t‐test. The *p* values in panels (g) and (i) were calculated using one‐way ANOVA. *p < 0.05; **p < 0.01; ***p < 0.001.

To explore the role of SNRPA in AS and the mechanisms of SNRPA‐induced DNA repair, we performed a genomic structure analysis based on transcriptome sequencing results. This analysis identified 1416 significant endogenous AS events regulated by SNRPA, with significant changes in percent spliced in (PSI) values (absolute ΔPSI ≥ 15%, *P* < 0.05) (**Figure** **4**a; Table S2, Supporting Information). SNRPA regulates various AS types, including intron retention (IR), alternative 5′ splice sites (A5SS), alternative 3′ splice sites (A3SS), mutually exclusive exons (MXE), and exon skipping (SE). The predominant AS event was SE (N = 1185), with 681 SE events showing increased exon skipping (∆PSI ≥ +15%) and 504 SE events exhibiting decreased exon skipping (∆PSI ≤ −15%) (Figure 4a,b). To identify the AS events regulating DNA repair, we intersected genes that underwent AS with those related to DNA repair (GOBP_DNA_REPAIR)(Table S3, Supporting Information). We found 67 alternative splicing genes, which involve 98 alternative splicing events, related to DNA repair (Figure 4c). Among these AS events, ERCC1 isoforms with deletion of exon VIII have been reported to have increased cisplatin sensitivity compared to the wild type.^[^ ^33^ ^]^ Encouragingly, SNRPA knockout led to an increased exclusion of exon 8, generating more ERCC1 transcripts with exon 8 exclusion [ERCC1‐E8(−)] and fewer ERCC1 transcripts with exon 8 inclusion [ERCC1‐E8(+)] compared with the NC group (Figure 4d). The proportion of ERCC1‐E8(−) was elevated after SNRPA knockout (Figure 4e). Specific primers targeting exon 7 and exon 9, which span exon 8, for RT‒PCR were designed to distinguish whether exon 8 was included or excluded (Figure 4f). The findings of the genomic structure analysis were validated via agarose gel electrophoresis. SNRPA depletion resulted in an increased ratio of ERCC1‐E8(−) expression and a decreased ratio of ERCC1‐E8(+) expression, which is referred to as the spliced‐out ratio [SOR, i.e., ERCC1‐E8(−)/ERCC1‐E8(+)] (Figure 4g,i), whereas the overexpression of SNRPA elicited the opposite effect, significantly reducing the SOR of ERCC1‐E8 (Figure 4h,j). In summary, SNRPA promotes alternative splicing of the ERCC1 precursor mRNA to form ERCC1‐E8(+).

ERCC1 forms a heterodimer with XPF, which is a structure‐specific endonuclease that is vital for DNA damage repair.^[^ ^34^ ^]^ The wild‐type long ERCC1 gene structure consists of 10 exons and encodes 297 amino acids (Figure 4k). The wild‐type elongated ERCC1 protein is composed of five primary α‐helical structures, designated H1 to H5. The H1 and H2 helices collectively form the initial HhH motif, whereas the second HhH motif is composed of the H4 and H5 helices.^[^ ^15^ ^]^ The two HhH motifs are XPF protein‐binding domains. ERCC1 residues Arg234 to Leu294 are considered indispensable binding sites for XPF.^[^ ^16^ ^]^ Through the National Center for Biotechnology Information (NCBI), we found that exon 8 of the ERCC1 gene encodes residues Ser235 to Gly258, which are in the first HhH motif of the XPF protein‐binding domain (Figure 4k). A reasonable assumption is that the upregulated exon VIII‐deficient ERCC1 variant attenuates the excision repair capacity of ERCC1 and augments the susceptibility of cancer cells to cisplatin treatment.^[^ ^33^ ^]^ As expected, SNRPA depletion markedly reduced the recruitment of XPF to ERCC1 (Figure 4l). Conversely, SNRPA overexpression promoted the interaction between XPF and ERCC1 (Figure 4m).

### 2.5 ERCC1‐E8(+) is Essential for SNRPA‐Modulated Cisplatin Resistance and DNA Repair

Cells employ the ERCC1–XPF complex to repair DNA damage.^[^ ^34^ ^]^ Given that the amino acids encoded by exon 8 of ERCC1 are involved in the interaction between ERCC1 and XPF, we investigated whether SNRPA‐induced cisplatin resistance and DNA repair depended on exon 8. First, siRNAs targeting ERCC1‐E8(+) (siE8‐1 and siE8‐2) were transfected into cisplatin‐resistant cells. Through agarose gel electrophoresis assays, we confirmed the successful silencing of ERCC1‐E8(+) in A549/DDP and H1299/DDP cells (Figure S6a,b, Supporting Information). Next, we discovered that ERCC1‐E8(+) knockdown did not influence the proliferation and apoptosis of chemotherapy‐resistant LUAD cells without cisplatin treatment (Figure S6c,d, Supporting Information). The results of the CCK‐8 experiments assessing cisplatin cytotoxicity demonstrated a significant reversal of cisplatin resistance in A549/DDP and H1299/DDP cells upon the silencing of ERCC1‐E8(+) compared with that in the control group (Figure S7a, Supporting Information). The results of the colony formation (Figure S7b, Supporting Information) and EdU (Figure S7c,d, Supporting Information) assays indicated that the knockdown of ERCC1‐E8(+) significantly reduced the survival and proliferation of A549/DDP and H1299/DDP cells treated with cisplatin. The flow cytometry analysis of apoptosis and Western blot analysis of apoptotic markers determined that ERCC1‐E8(+) silencing promoted apoptosis (Figure S7e–g, Supporting Information). Consistently, Western blot analysis showed a significant increase in the expression levels of the DNA damage biomarker *γ*‐H2AX in cells after the knockdown of ERCC1‐E8(+) (Figure S7g, Supporting Information). These results underscore the crucial role of the ERCC1‐E8(+) isoform in promoting cisplatin resistance and DNA repair in LUAD cells.

**Figure 5:**
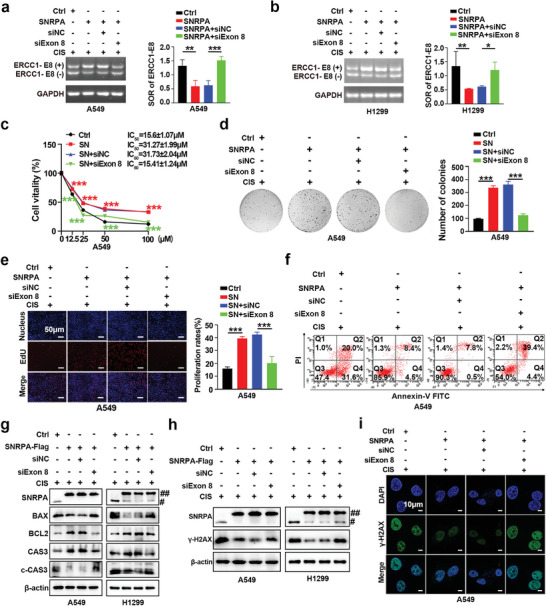
ERCC1‐E8(+) is critical for SNRPA‐orchestrated cisplatin resistance and DNA damage repair. a,b) Agarose gel showing the SOR of ERCC1‐E8 in cisplatin‐resistant A549 and H1299 cells after ERCC1‐E8(+) knockdown (siExon 8 or siE8‐1, siE8‐2) and SNRPA overexpression. Cropped blots are shown for the indicated ERCC1 isoforms or GAPDH. For uncropped blots, see Supporting Information. c) The viability of the designated cells was quantified utilizing a CCK‐8 assay following 48 h of exposure to cisplatin (n = 4). d) The specified cells were subjected to 14 days of treatment with 10 µM cisplatin, as assessed by a colony formation assay. Staining of the resultant colonies were stained with crystal violet (left panel), and a quantitative analysis of the colony frequency is presented in the bar graphs (right panel). e) An EdU incorporation assay was employed to measure the proliferation rates of the cells of interest under treatment with a 10 µM dose of cisplatin. f) The left panel displays representative images of Annexin V‐FITC/PI staining of the specified cells following 24 h of treatment with 10 µM cisplatin. g,h) Western blots showed the expression of apoptosis‐associated proteins and *γ*‐H2AX, a known indicator of DNA damage and repair; ## represents exogenous Flag‐SNRPA, and # represents endogenous SNRPAs. i) Co‐localization of *γ*‐H2AX and DAPI immunofluorescence revealed *γ*‐H2AX nuclear foci. All data are presented as the mean ± SD (n ≥ 3). The *p* values in panels (a), (b), (d) and (e) were calculated using one‐way ANOVA. The *p* values in panels (c) were calculated using two‐way ANOVA. *p < 0.05; **p < 0.01; ***p < 0.001.

The overexpression of SNRPA decreased the SOR of ERCC1‐E8 in A549 and H1299 cells (**Figure** **5**a,b). Consistent with our expectations, the silencing of ERCC1‐E8(+) significantly reversed SNRPA‐enhanced cisplatin resistance, as evidenced by CCK‐8 (Figure 5c; Figure S8a, Supporting Information), colony formation (Figure 5d; Figure S8b, Supporting Information), EdU (Figure 5e; Figure S8c, Supporting Information) and apoptosis‐related (Figure 5f,g; Figure S8d, Supporting Information) assays. Additionally, SNRPA overexpression enhanced DNA repair, contributing to the reduced presence of *γ*‐H2AX foci, whereas the silencing of ERCC1‐E8(+) led to increased expression of *γ*‐H2AX (Figure 5h,i; Figure S8e, Supporting Information). These results underscore the crucial role of exon 8 of ERCC1 in SNRPA‐induced cisplatin resistance in LUAD cells.

### 2.6 The Stability of the SNRPA mRNA, which is Modified by m^6^A Methylation, is Regulated by the ELAVL1 Protein

**Figure 6:**
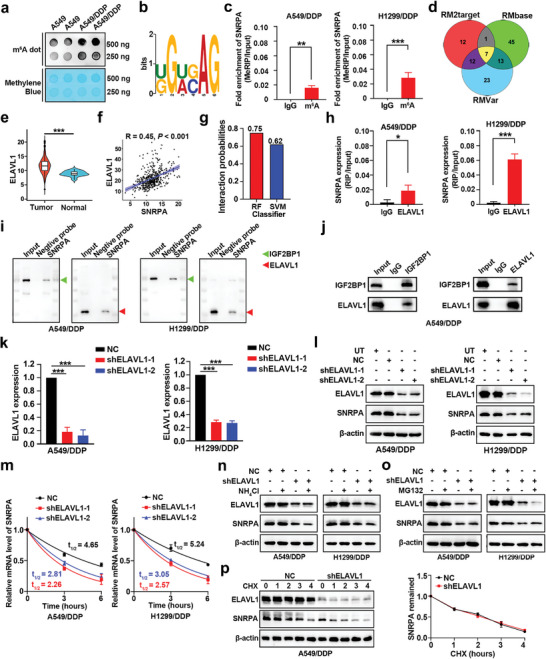
SNRPA expression and mRNA stability are regulated by ELAVL1. a) Dot blot assays illustrated the global m^6^A level in the total RNA of cisplatin‐resistant LUAD cell lines and their corresponding parental counterparts. b) The typical m^6^A motif RRACH (D = A, G or U; R = A or G; H = A, U or C) is shown in the SNRPA mRNA using MEME. c) The bar graphs showed that the m^6^A modification occurred in SNRPA mRNA, as determined via MeRIP‐qPCR. Anti‐m6A: m6A antibody. d) Venn diagram illustrating the m^6^A regulators controlling SNRPA, which were predicted by RM2target, RMbase, and RMVar. e) The expression levels of the ELAVL1 gene in tumor and normal tissues obtained from TCGA‐LUAD database were depicted using a violin plot. f) Pearson's correlation analysis of ELAVL1 and SNRPA expression in TCGA‐LUAD dataset. g) The probability of ELAVL1 binding to SNRPA was predicted by RPISeq. RF: random forest algorithm. SVM: Support vector machine algorithm. h) RIP‐qPCR revealed that the SNRPA mRNA was enriched by the ELAVL1 antibody. i) RNA pulldown analysis revealed the immunoprecipitation of ELAVL1 and IGF2BP with biotin‐labeled SNRPA probes. Red arrows: ELAVL1; green arrows: IGF2BP1. j) Immunoprecipitation assays confirmed the molecular interaction between IGF2BP1 and ELAVL1. k,l) qRT‒PCR and Western blot analyses revealed that the depletion of ELAVL1 (shELAVL‐1 and shELAVL‐2) resulted in a subsequent decrease in SNRPA expression in A549/DDP and H1299/DDP cells. m) Using an actinomycin D (Act D) pulse‐chase experiment, the half‐lives of the SNRPA mRNA in the indicated cells treated with 5 µg mL^−1^ Act D were observed. n,o) Western blot was utilized to evaluate the protein expression levels of ELAVL1 and SNRPA in targeted cells treated with the lysosome inhibitor NH_4_Cl (left panel) or the proteasome inhibitor MG132 (right panel), each for a period of 8 h. p) The stability of the SNRPA protein displayed by Western blot in LUAD cells treated with CHX in different time gradients. All data are presented as the mean ± SD (n = 3). The *p* values in panels (c), (e) and (h) were calculated by Student's t‐test. The *p* values in panels (k) were calculated using one‐way ANOVA. The *p* values in panels (m) were calculated using two‐way ANOVA. *p < 0.05; **p < 0.01; ***p < 0.001.

m^6^A RNA methylation is considered to facilitate cisplatin resistance.^[^ ^35^ ^]^ m^6^A dot plot assays demonstrated elevated levels of overall m^6^A methylation in the cisplatin‐resistant LUAD cell lines as opposed to their parental counterparts (**Figure** **6**a). Furthermore, using the online bioinformatic tool MEME (https://meme‐suite.org/meme/tools/meme), the typical m^6^A motif DRACH (D = A, G or U; R = A or G; H = A, U or C) was identified in the SNRPA mRNA (Figure 6b). To test the prediction of the presence of the SNRPA mRNA m^6^A methylation, methylated RNA immunoprecipitation (MeRIP) for the enrichment of methylated SNRPA was performed (Figure S9a, Supporting Information). MeRIP‐qPCR experiments revealed that, compared with the IgG control, SNRPA exhibited significant m^6^A methylation in LUAD cells (Figure 6c). Using the RMVar (https://rmvar.renlab.org/), RM2target (http://rm2target.ca nceromics.org/), and RMbase (https://rna.sysu.edu.cn/rmbase/index.php) online databases, we predicted seven m^6^A regulators that might be involved in SNRPA mRNA m^6^A methylation (Figure 6d). Among them, ELAVL1 [also known as human antigen R (HuR)] was most significantly overexpressed in LUAD tissues and had the lowest *p* value compared with normal lung tissues (Figure 6e; Figure S9b,c, Supporting Information). Pearson's correlation analysis revealed that the most significant correlation was observed between the mRNA expression levels of SNRPA and ELAVL1 among the seven m^6^A regulators (R = 0.45; *p* < 0.001; Figure 6f; Figure S9d,e, Supporting Information).

Recent studies have indicated that ELAVL1 can bind to mRNA, thereby increasing their stability.^[^ ^36^, ^37^ ^]^ The online tool RPISeq (http://pridb.gdcb.iastate.edu/RPISeq/) was used to predict potential interactions between the ELAVL1 protein and SNRPA mRNA (Figure 6g). RNA binding protein immunoprecipitation (RIP) and qRT‒PCR assays demonstrated that the SNRPA mRNA was greatly enriched by the ELAVL1 antibody (Figure 6h; Figure S10a, Supporting Information). IGF2BP proteins recognize m^6^A‐modified mRNAs, recruiting ELAVL1 as an RNA stabilizer to prevent mRNA degradation.^[^ ^38^ ^]^ RNA‐binding protein pull‐down assay (RNA pull‐down) assays followed by Western blot analyses were conducted to further confirm the interaction between ELAVL1 and the SNRPA mRNA, as well as between IGF2BP and the SNRPA mRNA. The results revealed that the ELAVL1 and IGF2BP proteins were pulled down by the biotinylated SNRPA mRNA probes (Figure 6i). The association between ELAVL1 and IGF2BP was confirmed through IP assays. IGF2BP was identified in the complex that precipitated with ELAVL1, and reciprocally, ELAVL1 was subsequently detected in the IP of IGF2BP (Figure 6j).

qRT‒PCR and Western blot analyses revealed that ELAVL1 depletion (shELAVL1‐1 and shELAVL1‐2) resulted in a subsequent decrease in SNRPA expression (Figure 6k,l). Next, we wondered whether the ELAVL1‐mediated effect on SNRPA expression occurs at the mRNA or protein level. An actinomycin D (Act D) chase assay revealed that silencing ELAVL1 diminished the stability of the SNRPA mRNA in LUAD cells (Figure 6m). However, the proteasome inhibitor MG132 and the lysosome inhibitor NH_4_Cl did not reverse the reduction in the SNRPA protein level elicited by ELAVL1 silencing (Figure 6n,o). In addition, ELAVL1 knockdown did not affect the half‐life of SNRPA following treatment with actinomycin (CHX) (Figure 6p; Figure S11a, Supporting Information). Together, our data suggest that ELAVL1 binds to the SNRPA mRNA, promoting its stability through the m^6^A modification and increasing SNRPA expression.

### 2.7 SNRPA is Essential for the Increase in Cisplatin Resistance, DNA Repair, and ERCC1‐E8(+) Expression Mediated by ELAVL1

The roles and mechanisms of ELAVL1 in cisplatin resistance are unknown. Without cisplatin treatment, ELAVL1 knockdown inhibited cell viability but promoted apoptosis and DNA damage in chemotherapy‐resistant LUAD cells (Figure S10, Supporting Information). Cisplatin did not inhibit cell proliferation or induce apoptosis in chemotherapy‐resistant LUAD cells, however the combination of ELAVL1 silencing and DNA damage cisplatin showed a more significant inhibition of cell viability and a greater promotion of apoptosis and DNA damage than either cisplatin or shRNA‐ELAVL1 alone (Figure S10, Supporting Information).

**Figure 7:**
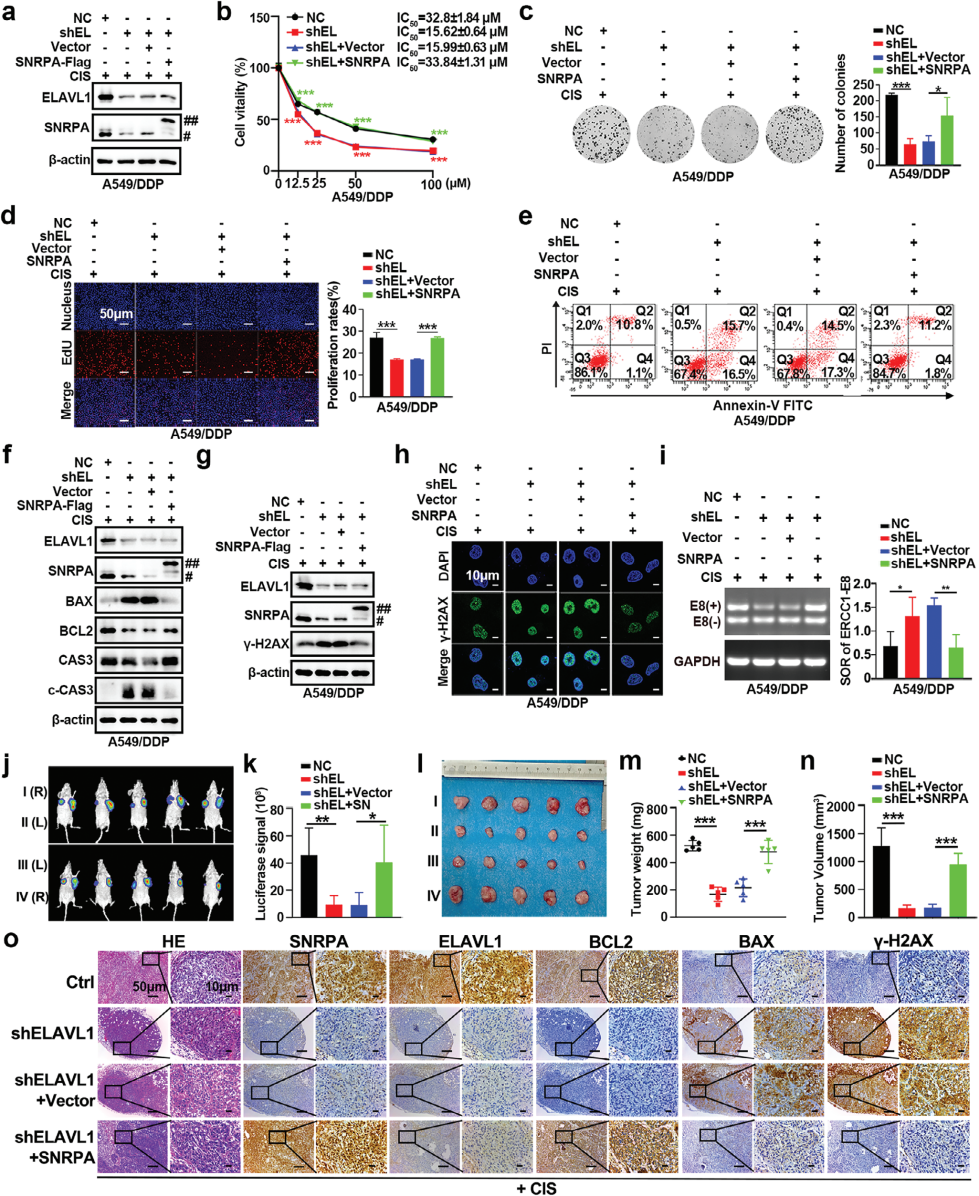
ELAVL1 promotes cisplatin resistance, DNA repair and ERCC1‐E8(+) expression in an SNRPA‐dependent manner. a) Western blot analysis was utilized to determine the levels of ELAVL1 and SNRPA expression in cisplatin‐resistant cell lines following the overexpression of SNRPA based on the knockdown of ELAVL1; ## represents exogenous Flag‐SNRPA, and # represents endogenous SNRPA. b) Cell survival in the defined populations was quantified after 48 h of cisplatin exposure using CCK‐8 assays (n = 4). c) Colonies were visualized with crystal violet staining (left panel), and a quantitative assessment of colony numbers was presented in the accompanying bar graphs for statistical interpretation (right panel). d) EdU assays showing the proliferation of the indicated cells in a 10 µM cisplatin solution. e) The left panel presents micrographs illustrating the staining of the designated cells with Annexin V‐FITC/PI following exposure to 10 µM cisplatin for 24 h. f) The expression levels of proteins associated with apoptosis were analyzed using Western blot; ## represents exogenous Flag‐SNRPA, and # represents endogenous SNRPA. g) Western blot was used to assess the expression of *γ*‐H2AX, a well‐known indicator of DNA damage and repair; ## represents exogenous Flag‐SNRPA, and # represents endogenous SNRPA. h) Immunofluorescence co‐localization of *γ*‐H2AX and DAPI in A549/DDP cells. i) Agarose gel showing that SNRPA overexpression effectively restored the ELAVL1 knockdown‐mediated downregulation of ERCC1‐E8(+). Cropped blots are shown for the indicated ERCC1 isoforms or GAPDH. For uncropped blots, see Supporting Information. j‐n) Athymic nude mice were allocated into four experimental cohorts: the NC, shELAVL1 (shEL), shELAVL1+Vector (shEL+Vector), and shELAVL1+SNRPA‐Flag (shEL+SNRPA) cohorts. j,k) Bioluminescence images of xenograft tumors were shown. The bioluminescent signals were quantified and are presented in bar graphs. Additionally, at the culmination of the experiment, photographic documentation of the xenograft tumors from the 4 groups was performed (l). Subsequent statistical assessments were conducted to compare the mean tumor weights (m) and volumes (n) across the groups. o) IHC analyses were carried out to determine the protein expression levels within the tumors. All data are presented as the mean ± SD (n ≥ 3). The *p* values in panels (d), (i), (k), (m) and (n) were calculated using one‐way ANOVA. The *p* values in panels (b) were calculated using two‐way ANOVA. *p < 0.05; **p < 0.01; ***p < 0.001.

As shown in **Figures** **7**a and S12a (Supporting Information), upon ELAVL1 knockdown, SNRPA was overexpressed. Indeed, after ELAVL1 silencing, the resistance of A549/DDP and H1299/DDP cells was reversed (Figure S11b–e, Supporting Information), apoptotic cells were markedly increased with cisplatin treatment (Figure S11f,g, Supporting Information). Moreover, ELAVL1 depletion inhibited DNA repair (Figure 7g,h; Figure S12g, Supporting Information) and ERCC1‐E8(+) expression in the presence of cisplatin (Figure 7i; Figure S12i, Supporting Information). Consistent with our expectation, SNRPA overexpression effectively restored the ELAVL1 knockdown‐mediated reduction in cisplatin resistance and DNA repair and the downregulation of ERCC1‐E8(+) (Figure 7a–i; Figure S12, Supporting Information). To ascertain compelling in vivo evidence for the indispensability of SNRPA in ELAVL1‐mediated augmentation of cisplatin resistance, athymic mice were stratified into the following four cohorts: (I) NC, (II) shELAVL1, (III) shELAVL1+Vector, and (IV) shELAVL1+SNRPA. Relative to athymic mice inoculated with NC cells, those inoculated with shELAVL1 cells exhibited a reduced tumor burden (Figure 7j–n), intimating that ELAVL1 may potentiate cisplatin resistance. Compared with the third group (III), the fourth group (IV) exhibited an increased tumor size (Figure 7j–n). IHC assays revealed elevated levels of BAX and *γ*‐H2AX in group (II) than in group (I), whereas the expression of BCL2 was markedly diminished (Figure 7o). SNRPA overexpression counteracted the effects of ELAVL depletion on cisplatin sensitivity and DNA damage repair, as evidenced by decreased BAX and *γ*‐H2AX expression and increased BCL2 expression (Figure 7o). Our results support the conjecture that ELAVL1 may promote cisplatin resistance in an SNRPA‐dependent manner.

### 2.8 SNRPA Expression is Correlated with Therapeutic Resistance to Cisplatin in Clinical LUAD Specimens

**Figure 8:**
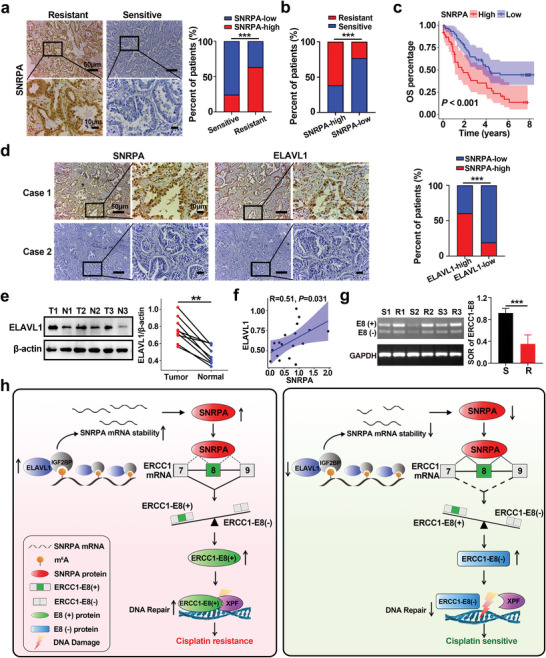
Correlations between the expression levels of SNRPA and ELAVL1 in tissues obtained from individuals diagnosed with LUAD. a,b) The left panel shows the results of IHC staining for SNRPA in LUAD tissues, which were categorized into cisplatin‐sensitive (PFS ≥ 6 months) and cisplatin‐resistant (PFS < 6 months) subgroups. The right panel quantifies the frequency of high (red bar) versus low (blue bar) SNRPA expression correlated with cisplatin responsiveness. b) This bar graph delineates the distribution of cisplatin‐resistant (red bar) versus cisplatin‐sensitive (blue bar) patients in the high‐ and low‐SNRPA expression cohorts. c) Kaplan–Meier survival plots delineating the OS stratification for 100 patients treated with cisplatin from Harbin Medical University Cancer Hospital. d) Consecutive LUAD tissue sections from two individuals display the IHC staining intensity for ELAVL1 and SNRPA. Case 1 correlates with high SNRPA expression, while Case 2 aligns with low SNRPA expression in LUAD tissues. e) Western blot revealed the protein expression of ELAVL1 using fresh frozen LUAD (n = 9) and normal adjacent tissues (n = 9), the others could be seen in Figure S13a (Supporting Information). f) Correlation between the expression of ELAVL1 and SNRPA based on Western blot results from nine pairs of fresh frozen tissues. g) Agarose gel electrophoresis showing the expression of ERCC1‐E8 (+) and ERCC1‐E8 (−) in fresh frozen cisplatin‐sensitive (S) (n = 9) and cisplatin‐resistant (R) (n = 9) tissue samples (left panel). Cropped blots are shown for the indicated ERCC1 isoforms or GAPDH. For uncropped blots, see Supporting Information. The bar graph shows the spliced‐out ratio (SOR) of ERCC Exon 8 (right panel). SOR: ERCC1‐E8 (−)/ERCC1‐E8 (+). Additional data can be seen in Figure S13b (Supporting Information). h) A schematic representation illustrating the molecular mechanism by which m^6^A‐modified SNRPA, which regulates the AS of ERCC1, contributes to the development of cisplatin resistance. All data are presented as the mean ± SD (n ≥ 3). The *p* values in panels (e) were calculated by Student's t‐test. The *p* values in panels (a), (b) and (d) were calculated by Chi‐square test. **p < 0.01; ***p < 0.001.

To investigate the association between SNRPA expression and cisplatin resistance, LUAD specimens from patients who had received cisplatin treatment were selected. IHC results showed that cisplatin‐resistant patients (PFS < 6 months) exhibited higher SNRPA expression levels than cisplatin‐sensitive patients (PFS ≥ 6 months **Figure** **8**a). A greater proportion of cisplatin‐resistant patients exhibited elevated levels of SNRPA expression in comparison to cisplatin‐sensitive patients (Figure 8b). Kaplan–Meier analysis based on 100 LUAD patients treated with cisplatin demonstrated that high SNRPA expression predicted poor overall survival (Figure 8c). The relationship of the intensity and distribution between SNRPA and ELAVL1 was positive, suggesting that ELAVL1 might upregulate SNRPA expression (Figure 8d). Using fresh frozen LUAD (T) and normal adjacent (N) tissue samples, we found that the expression of ELAVL1 was upregulated in LUAD tissue compared with the normal tissue (Figure 8e; Figure S13a, Supporting Information). Moreover, the spearman correlation suggests that there was a significant positive correlation between the protein expression of ELAVL1 and SNRPA (Figure 8f). Meanwhile, ERCC1‐E8 (+) expression were significantly higher in resistant patient tissues compared to sensitive tissues, as indicated by the lower spliced‐out ratio (SOR), i.e., ERCC1‐E8 (−)/ERCC1‐E8 (+), of ERCC Exon 8 (Figure 8g; Figure S13b, Supporting Information). These findings, combined with the experimental data derived from LUAD cell lines and xenograft models, reveal that m^6^A‐driven SNRPA can contribute to cisplatin resistance through SNRPA‐mediated apoptosis and DNA repair.

## 3 Discussion

The precise role and underlying mechanisms of AS in conferring resistance to cancer treatments, particularly cisplatin chemotherapy in LUAD, remain elusive. Our study demonstrated that SNRPA is upregulated in cisplatin‐resistant LUAD cells, which augments cellular chemoresistance to cisplatin by regulating the splicing of exon 8 of the ERCC1 gene. Specifically, the upregulation of ELAVL1 promotes reading of m^6^A methylation and increases SNRPA mRNA stability and expression. This increased SNRPA level then promotes the expression of ERCC1‐E8(+), which includes exon 8 and is responsible for coding the XPF protein‐binding domain. The resultant upsurge in the production of the ERCC1‐XPF complex leads to enhanced DNA repair and a subsequent enhancement of cisplatin resistance (Figure 8h).

In this study, SNRPA was identified for the first time as a factor related to sensitivity to platinum‐based drugs. The rapid advancement of next‐generation high‐throughput RNA sequencing technologies has resulted in the production of tremendous amounts of high‐dimensional “omics” data.^[^ ^39^ ^]^ Owing to the vast and reliable data‐sharing efforts of research communities, such as those by TCGA, a wealth of diverse “omics” data, characterized by an unprecedented level of detail from adequately large cohorts, are accessible.^[^ ^40^ ^]^ Survival analyses were conducted on both the treated and untreated groups using these public datasets to explore the associations between tumor‐related factors and therapeutic sensitivity to radiotherapy and chemotherapy.^[^ ^41^, ^42^ ^]^

SNRPA (also known as U1A) is a core component of the U1 snRNP, which is one of the five essential snRNPs of the spliceosome.^[^ ^43^ ^]^ The U1 snRNP facilitates the recognition and binding of the 5′ splice site of pre‐mRNA, a critical step in the splicing reaction.^[^ ^44^ ^]^ The SNRPA protein is characterized by its RNA recognition motif (RRM), which is instrumental in mediating interactions with RNA substrates.^[^ ^45^ ^]^ Given its central role in mRNA splicing, SNRPA has been implicated in numerous diseases, including cancer.^[^ ^46^, ^47^ ^]^ Elevated expression of SNRPA correlates with an unfavorable prognostic outcome in patients with LUAD,^[^ ^48^ ^]^ hepatocellular carcinoma,^[^ ^49^ ^]^ and cervical cancer.^[^ ^50^ ^]^ SNRPA augments neoplastic cell proliferation in gastric carcinoma by modulating the expression of nerve growth factor.^[^ ^51^ ^]^ SNRPA promotes the proliferation and migration while inhibiting the apoptosis of hepatocellular carcinoma cells.^[^ ^49^ ^]^ The association of SNRPA with microvascular invasion is implicated in the promotion of metastatic progression in hepatocellular carcinoma via the activation of the NOTCH1/Snail signaling cascade.^[^ ^52^ ^]^ In this study, we observed that SNRPA has no effect on the proliferation and apoptosis of LUAD cells without cisplatin. This discrepancy, unlike what is seen in gastric carcinoma and hepatocellular carcinoma, might be due to the unique molecular and genetic landscape of LUAD cells. LUAD is characterized by distinct oncogenic mutations (such as EGFR mutations) that co‐occur with many tumor suppressor gene alterations, which could alter the way SNRPA interacts with cellular machinery.^[^ ^53^ ^]^ Additionally, the tumor microenvironment in LUAD, with its specific extracellular matrix components and signaling molecules, might also contribute to this differential regulation.^[^ ^54^ ^]^

In this study, SNRPA was first identified as a regulator of the ERCC1 exon 8 skipping event. The SNRPA protein, comprising 282 amino acids with an approximate molecular mass of 34 kilodaltons, is characterized by the presence of two RNA recognition motifs (RRM).^[^ ^55^ ^]^ Indeed, SNRPA is capable of interacting with the loop region of the SL2 sequence AUUGCAC within the U1 RNA, facilitated by its N‐terminal RNA recognition motif.^[^ ^56^ ^]^ SNRPA possesses the capacity to associate with the G‐quadruplex structure within the 5′ untranslated region of BAG‐1, and knockdown studies in colorectal cancer cell lines have indicated that SNRPA may exert a regulatory influence on the expression of BAG‐1.^[^ ^57^ ^]^ The potential interaction between SNRPA and the ERCC1 pre‐mRNA, including determination of the specific RNA recognition motif of SNRPA, as well as the corresponding sequence within ERCC1, is interesting to be elucidated in the future.

In this study, SNRPA was shown to regulate the formation of the ERCC1 and XPF complex. SNRPA governs the alternative splicing of exon 8 within the ERCC1 gene, encoding amino acids from serine 235 to glycine 258. This region corresponds to the initial HhH motif in the ERCC1 protein, a crucial domain for binding to the XPF protein (Figure 4k).^[^ ^16^ ^]^ The ERCC1‐XPF heterodimer functions as an essential endonuclease within the NER pathway, facilitating the excision of helix‐distorting DNA lesions.^[^ ^58^ ^]^ ERCC1 serves primarily in damage recognition and repair complex assembly processes, whereas XPF provides the enzymatic activity required for excising the damaged DNA strand.^[^ ^59^ ^]^ In addition to NER, the ERCC1‐XPF complex is involved in other DNA repair pathways, such as HR and ICL, highlighting the multifaceted role of this complex in maintaining genomic integrity.^[^ ^60^, ^61^ ^]^ Their coordinated actions are critical for maintaining genomic stability by ensuring the accurate and efficient repair of a broad spectrum of DNA lesions, including those induced by cisplatin.^[^ ^61^ ^]^ Consistent with our findings, targeted inhibition of the structure‐specific endonuclease function of ERCC1‐XPF potentiated cisplatin resistance.^[^ ^62^, ^63^ ^]^ The overexpression of an exon VIII‐deficient variant of ERCC1 attenuated the NER capacity of ERCC1 and augmented the susceptibility of these cells to cisplatin in a dose‐dependent manner.^[^ ^33^ ^]^

ELAVL1, also known as HuR, is a pivotal RNA‐binding protein that modulates the posttranscriptional fate of a myriad of mRNAs bearing AU‐rich elements in untranslated regions (UTRs).^[^ ^64^ ^]^ As an RNA stabilizer, ELAVL1, in conjunction with IGF2BP, which recognizes m^6^A modifications in transcripts, stabilizes these transcripts by preventing their degradation.^[^ ^38^ ^]^ For instance, the recruitment of HuR to m^6^A‐modified sites is imperative for the stabilization of SOX2 messenger RNA mediated by methyltransferase‐like protein 3 (METTL3).^[^ ^65^ ^]^ The m^6^A modification of the ZMYM1 mRNA by METTL3 augmented its stability through a mechanism dependent on ELAVL1.^[^ ^66^ ^]^ In the context of cisplatin resistance, ELAVL1 is capable of interacting with the ARID1A mRNA to increase its stability, which leads to the repression of DNA double‐strand break accumulation and increased chemoresistance to cisplatin.^[^ ^67^, ^68^ ^]^ Our data suggest a similar mechanism by which ELAVL1 can bind to the SNRPA mRNA and increase its stability, contributing to DNA repair and cisplatin resistance. Moreover, it is fascinating to investigate the presence of other epigenetic modifications on SNRPA, including DNA methylation, histone modification, chromatin remodeling, non‐coding RNA interactions, and various types of RNA methylation, such as m^1^A, m^5^C, and m^7^G.

## 4 Conclusion

In summary, we identified SNRPA as a spliceosome‐related gene that may modulate resistance to platinum chemotherapy. Our study not only provides insights into the biological functions and mechanistic insights into the role of SNRPA in cisplatin resistance but also uncovers a novel regulatory function of ELAVL1 in modulating chemotherapy sensitivity. These findings contribute to paving the way for personalized therapy targeting alternative splicing and the m^6^A modification.

## 5 Experimental Section

### Public Data Analysis and Clinical Tissue Samples

The Spliceosome database (http://spliceosomedb.ucsc.edu/) provides a synthesis of existing mass spectrometry data, revealing that 137 constituents of the spliceosome have been detected across various assembly stages of human spliceosomal complexes (Table S1, Supporting Information). Gene expression and clinical data were obtained from TCGA (https://portal.gdc.cancer.gov/) and the GEO(https://www.ncbi.nlm.nih.gov/geo/).

The screening criteria for differentially expressed genes (DEGs) were |log_2_FC| > 0.585 and *p* < 0.05. Differential expression heatmaps, survival analysis, and violin plots were generated using the R package. Independent prognostic factors were explored through Cox regression analyses and visualized in forest plots using the “survminer” R package. Additionally, the bioinformatics analysis workflow is illustrated in Figure S1 (Supporting Information).

Clinical tissue samples were collected from 100 LUAD patients who underwent surgery at Harbin Medical University Cancer Hospital.

### Cell Culture and Reagents

LUAD cell lines resistant to cis‐diammine‐dichloroplatinum (II) (A549/DDP and H1299/DDP) and cisplatin‐sensitive cells (A549 and H1299) were preserved in the laboratory,^[^ ^12^, ^69^ ^]^ and the resistance stability of these two cisplatin‐resistant cell lines was reconfirmed at the beginning of the study. The culture conditions were as follows: RPMI 1640 medium supplemented with 10% fetal bovine serum (FBS). Notably, A549/DDP and H1299/DDP cells were cultured with media containing 3 µM cisplatin (Biosharp, China).

The proteasome inhibitor MG132 (10 µM; HY‐13259, MedChemExpress) and the lysosome inhibitor NH_4_Cl (10 µM; 12125‐02‐9, Aladdin, China) were applied for 8 h in this study.

### Establishment of Stable Cell Lines

Guide RNAs (sgRNAs) were designed and validated by HanBio Technology (Shanghai, China), as outlined in Table S4 (Supporting Information). The pHBLV‐U6‐gRNA‐EF1‐CAS9‐PURO plasmid was engineered for this purpose. Upon sequencing confirmation of the plasmids, the constructs were transfected into A549/DDP cells. Following a 48‐h transfection period, the cells were screened with puromycin‐containing culture media (2 µg mL^−1^, Solarbio, CAS 58‐58‐2). Western blot and qRT‒PCR were employed to confirm the SNRPA knockout clones.

Short hairpin RNA (shRNA) oligonucleotides, sourced from GeneChem (Shanghai, China) and listed in Table S4 (Supporting Information), were employed to downregulate SNRPA in H1299/DDP cells and ELAVL1 in both A549/DDP and H1299/DDP cells. Lentiviruses encoding SNRPA overexpression sequences (SNRPA‐Flag/SNRPA) were generated using the Ubi‐SNRPA‐3FLAG‐SV40‐puromycin vector (GeneChem, Shanghai, China) for transfection. Following transfection, these cell lines were subsequently subjected to puromycin selection (2 µg mL^−1^).

### Transient Transfection

2 × 10^5^ cells were seeded in six‐well plates. After 24 h of incubation to allow cell attachment, a mixture consisting of 200 µL of buffer, 4 µL of Polyplus (jetPRIME transfection reagent), and 5 µL of siRNA (RiboBio) (refer to Table S4, Supporting Information) was applied to the plates. The cells were incubated for 6 h following the instructions of the transfection reagent, after which the mixture was replaced with complete medium for further incubation.

### Analysis of mRNA Expression

RNA was isolated using the Total RNA Kit I (R6834‐01; Omega Bio‐Tek). Subsequently, reverse transcription was carried out using the FastKing kit (KR118‐02; TIANGEN). qRT‒PCR was performed following the protocol provided with Talent qPCR Premix (FP209‐02, TIANGEN). A StepOne qRT‒PCR instrument (Applied Biosystems) was utilized to determine the expression levels of the target genes.

Splice variants were discriminated by electrophoresis. After the agarose gel was stained with GelRed dye (Biotium, 41003), the PCR products were added for electrophoresis. The signal was visualized with the FluorChem E system (ProteinSimple, CA, USA). The primer sequences are provided in Table S5 (Supporting Information).

### Western Blot

Following cell or LUAD tissue collection, proteins were extracted by adding RIPA lysis buffer (AR0102, BOSTER) supplemented with protease inhibitors, including PMSF (ST506, Beyotime). Standard procedures were followed for gel electrophoresis and protein transfer onto polyvinylidene difluoride (PVDF) membranes. The primary antibodies used are listed in Table S6 (Supporting Information).

### Resistance Assays

EdU incorporation, CCK‐8 and colony formation assays were used to assess viability, according to the previous studies.^[^ ^12^ ^]^ LUAD cells were seeded in the indicated plates after trypsin digestion. Following 24 h of cell adherence, the cells were treated with 10 µM cisplatin for an additional 24 h. For EdU incorporation, subsequent experimental steps were conducted according to the instructions provided with the EdU Assay Kit (C10310‐1; RiboBio, China). Fluorescence images were captured immediately after DNA staining. For the CCK‐8 assay, cell viability was then quantified based on absorbance measurements using a kit (CK04, Dojindo, Japan). For the colony formation assay, the LUAD cells were kept in culture medium supplemented with 5 µM cisplatin for 14 days. These colonies were then stained with crystal violet and photographed.

### RNA Sequencing (RNA‐seq) Analysis

An RNA‐seq transcriptome library was prepared using the Illumina Stranded mRNA Prep Ligation Kit (Illumina, USA). RNA transcriptome sequencing was employed to detect the differential expression of mRNAs between knockout (KO) and control (SNRPA‐NC) LUAD cells, with significance thresholds set at |log_2_FC| > 1 and a *p* value < 0.05. Data analysis, including alternative splice junctions, was conducted using the Majorbio Cloud Platform (Majorbio, Shanghai, China). An analysis of GO terms was performed using Apache ECharts (https://echarts.apache.org/zh/index.html).

### RNA m^6^A Dot Blot Assay

After total RNA was extracted with TRIzol reagent, the mRNA products were denatured for 3 min (95 °C) and transferred onto Hybond‐N+ membranes (Amersham, GE Healthcare, USA). Following UV crosslinking of the membranes for 25 min, they were blocked for 1 h with 5% skim milk powder. The m^6^A antibody was then applied and incubated overnight at 4 °C. A luminescent working solution was subsequently added for exposure and photography. Afterward, the membrane was stained with 0.02% methylene blue (G1301, Solarbio, China).

### Determining the m^6^A Regulators Targeting SNRPA

To identify potential m^6^A regulators involved in SNRPA mRNA methylation, the m^6^A WER Target Gene Database (RM2target, http://rm2target.canceromics.org/), the RNA Modification Base (RMBase, https://rna.sysu.edu.cn/rmbase/), and the Database of Functional Variants Involved in RNA Modifications (RMVAR, http://rmvar.renlab.org/) were utilized. The resulting dataset was analyzed for intersections using Venn diagrams. The relationships between SNRPA and m^6^A regulators were assessed by utilizing a bioinformatics website (http://www.bioinformatics.com.cn). Violin plots illustrating the expression of m^6^A regulators were generated using Hiplot (https://hiplot‐academic.com/basic/).

### MeRIP

Total RNA was harvested from LUAD cells, and it subsequently utilized the riboMeRIP m^6^A Transcriptome Profiling Kit (C11051‐1; RiboBio, China). The m^6^A‐modified RNAs were then analyzed via qPCR. SRAMP (http://www.cuilab.cn/sramp) was employed to predict the MeRIP‐qPCR primers for SNRPA.

### RIP

Cells were lysed on ice using RIP lysis buffer from an RNA immunoprecipitation kit (Bes5101, BersinBio, China). After centrifugation, the supernatant was collected and incubated with an anti‐IgG or m^6^A antibody. The immunoprecipitates were collected after the addition of magnetic beads, and the coprecipitated RNA was detected via qRT‒PCR.

### RNA Pull‐Down

The experiments were conducted using an RNA pull‐down kit (Bes5102, BersinBio, China). The biotin‐labeled full‐length SNRPA mRNA was synthesized by GenePharma. Lysates from A549/DDP and H1299/DDP cells were isolated and then incubated with the biotinylated SNRPA mRNA at 4 °C. Subsequently, streptavidin‐labeled magnetic beads were added to capture the complexes, which were then analyzed by Western blot after elution.

### RNA Stability Assay

The cells were treated with actinomycin D (5 µg mL^−1^) for 0, 3, or 6 h before being harvested and analyzed via qRT‒PCR. The data were normalized to the expression of GAPDH.

### Animal Experiments

All animal experiments were approved by the Ethics Committee of Harbin Medical University (Grant No. KY2023‐75). A total of 1 × 10^7^ tumor cells were injected subcutaneously into 4‐week‐old BALB/c mice (weighing 13–15 g). On the eighth day after injection, mice with detectable tumors (greater than or equal to 100 mm^3^) received cisplatin at a dosage of 5 mg kg^−1^ per mouse per administration. Injections were administered on days 8, 12, 16, 20, and 24, according to previous reports.^[^ ^70^, ^71^ ^]^ On the 28th day, in vivo animal fluorescence imaging was performed. After the mice were euthanized, the long and short diameters were determined using Vernier calipers and the tumor weights were measured.

### Statistical Analysis

The statistical analyses were executed using GraphPad Prism and SPSS Statistics software. Data were presented as the mean ± SD of at least three independent experiments. Differences were tested using the Student's t‐test, one‐way ANOVA, two‐way ANOVA, Fisher's exact test and Chi‐square test, with significance indicated when *p* < 0.05. Correlations were determined using Pearson's correlation analysis.

### Ethics

All patients in this study signed an informed consent form. This study was approved by the Ethics Committee of Harbin Medical University Cancer Hospital.

## Conflict of Interest

The authors declare no conflict of interest.

## Author Contributions

W.F., J.H., and F.T. contributed equally to this work. W.F., L.C., and Y.X. designed the study. W.F., J.H., and F.T. carried development of methodology. X.H., K.Z., Y.Z., X.L., X.W., and X.W. supported analysis and interpretation of data. W.F. conducted experiments. W.F., J.H., F.T., L.C., and Y.X. wrote, reviewed, and edited the manuscript. All authors read and approved the final manuscript.

## Supporting information



Supporting Information

Supplementary Tables

## Data Availability

The data that support the findings of this study are available in the Supporting Information of this article.
