# Supplementary material for: m6A‐Modified SNRPA Controls Alternative Splicing of ERCC1 Exon 8 to Induce Cisplatin Resistance in Lung Adenocarcinoma
Source: Adv Sci (Weinh). 2024 Nov 18;11(47):2404609. doi: 10.1002/advs.202404609 (PMC11653629; doi:10.1002/advs.202404609)
Supplement: Supplementary file 1 — Supporting Information [file ADVS-11-2404609-s002.docx]

Supporting Information

**m^6^A-modified SNRPA Controls Alternative Splicing of ERCC1 Exon 8 to Induce Cisplatin Resistance in Lung Adenocarcinoma**

*Weina Fan^1,^ ^2†^, Jian Huang^1†^, Fanglin Tian^1†^, Xin Hong^1^, Kexin Zhu^1^, Yuning Zhan^1^, Xin Li^1^, Xiangyu Wang^1^, Xin Wang^1^, Li Cai^1, 2*^, Ying Xing^1*^*


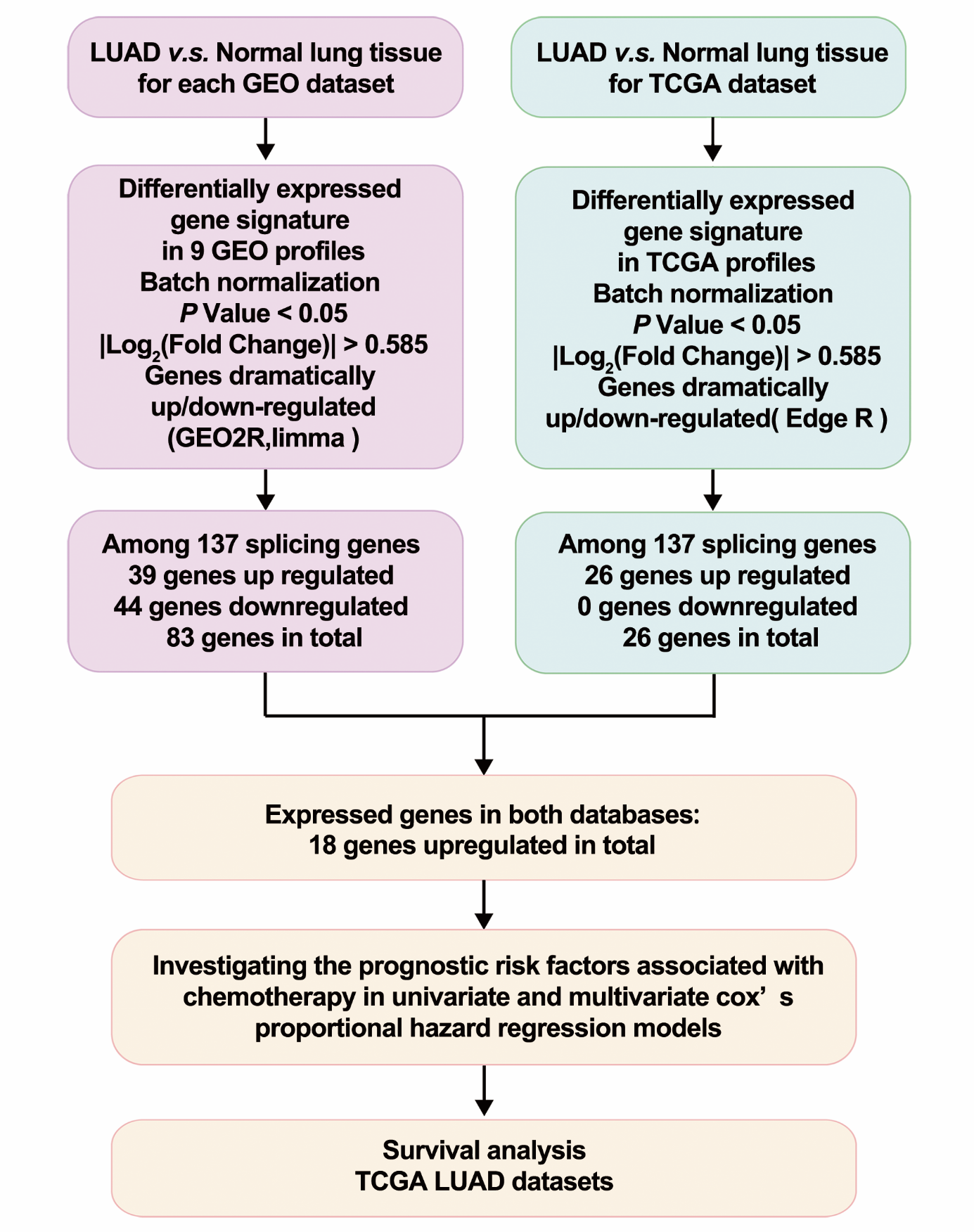


**Figure S1. Flowchart for the bioinformatics analysis used to identify spliceosome-related genes that might govern platinum-based drug resistance.**

**
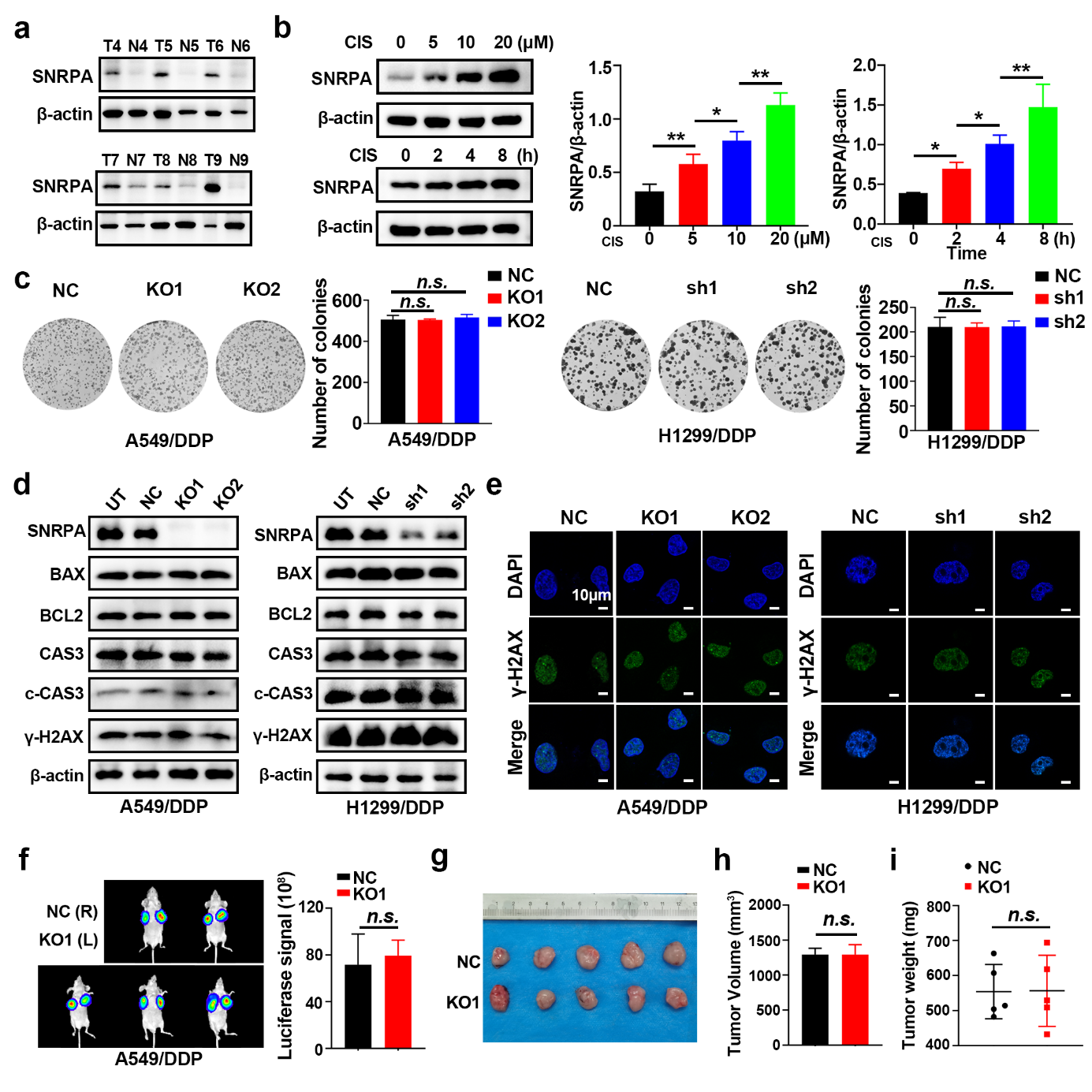
**

**Figure S2.** **SNRPA knockdown has no effect on the proliferation, apoptosis and DNA damage of LUAD cells without cisplatin.** a) Immunoblot analysis revealing SNRPA protein expression in fresh frozen LUAD and normal adjacent tissue samples. b) Western blot showing the levels of SNRPA expression after treatment with increasing cisplatin dosages or time durations of cisplatin treatment (left panel). The bar graphs showed the SNRPA protein expression according to the average grayscale value of the target protein (right panel). c) Cellular replication of delineated cell populations without cisplatin over 14 days was assessed via a colony formation assay. Colonies were visualized by staining with crystal violet (left panel), and bar graphs provided a statistical evaluation of the colony counts (right panel). d) The expression of apoptosis-associated proteins and γ-H2AX was determined. e) Immunofluorescence co-localization of γ-H2AX and DAPI. f) Bioluminescent imaging of xenograft tumors from NC or KO1 cells, was captured at 28 days without treatment of cisplatin, with the images of the harvested xenograft tumors. Statistical evaluation of the luciferase signal from the xenograft tumors is represented in bar graphs. g, i) Statistical analysis was conducted to determine the average tumor volume and weight among the different groups. Data are presented as the mean ± SD (n=3). The *P* values in panels (b) and (c) were calculated using one-way ANOVA. The *P* values in panels (f), (h) and (i) were calculated by Student’s t-test. **P* < 0.05; ***P* < 0.01; *n.s.* indicates no significance.

**
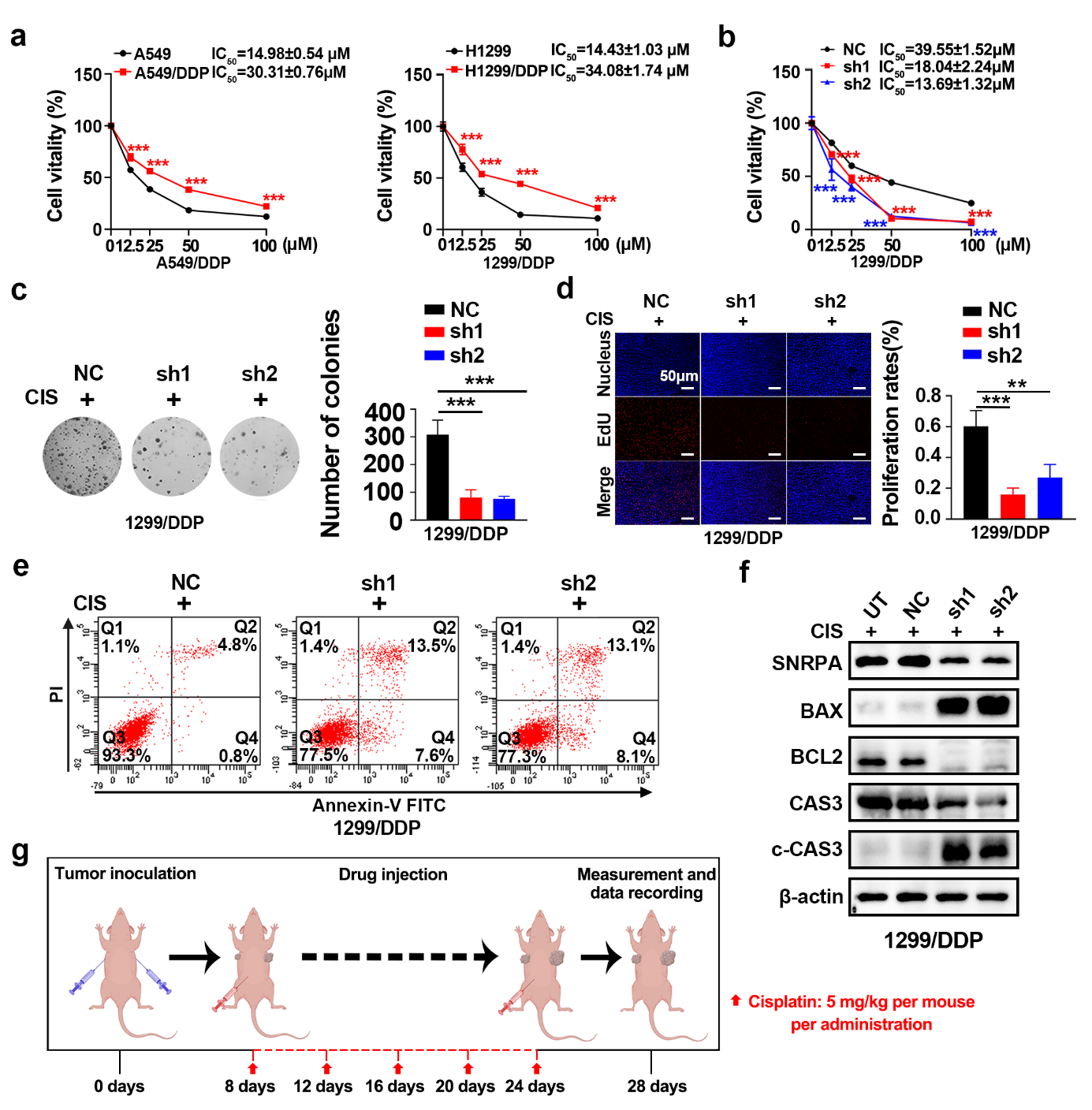
**

**Figure S3. SNRPA knockdown blunts the resistance of H1299/DDP cells to cisplatin.** a) The sensitivity of two types of cells, parental cisplatin-sensitive cells (A549 and H1299 cells) and cisplatin-resistant cells (A549/DDP and H1299/DDP cells), to cisplatin was evaluated (n=4). b) CCK-8 analysis of cell viability across the designated groups after 48 hours of cisplatin treatment (n=4). c) The delineated cell populations were administered 10 µM cisplatin over a period of 14 days, and the resultant effects on cellular replication assessed via a colony formation assay. Colony visualization was achieved by staining with crystal violet (left panel), while the bar graphs provided a statistical evaluation of the colony counts (right panel) (n=4). d) EdU assays showed the proliferation of the indicated cells in a 10 µM cisplatin solution. e) Representative images showing Annexin V-APC/7-AAD staining of the indicated cells following 24 hours of exposure to 10 µM cisplatin. f) The expression of apoptosis-associated proteins was determined. g) Design of subcutaneous tumor cell injections in nude mice. All data are presented as the mean ± SD (n ≥ 3). The *P* values in panels (c) and (d) were calculated using one-way ANOVA. The *P* values in panels (a) and (b) were calculated using two-way ANOVA. ***P* < 0.01; and ****P* < 0.001.


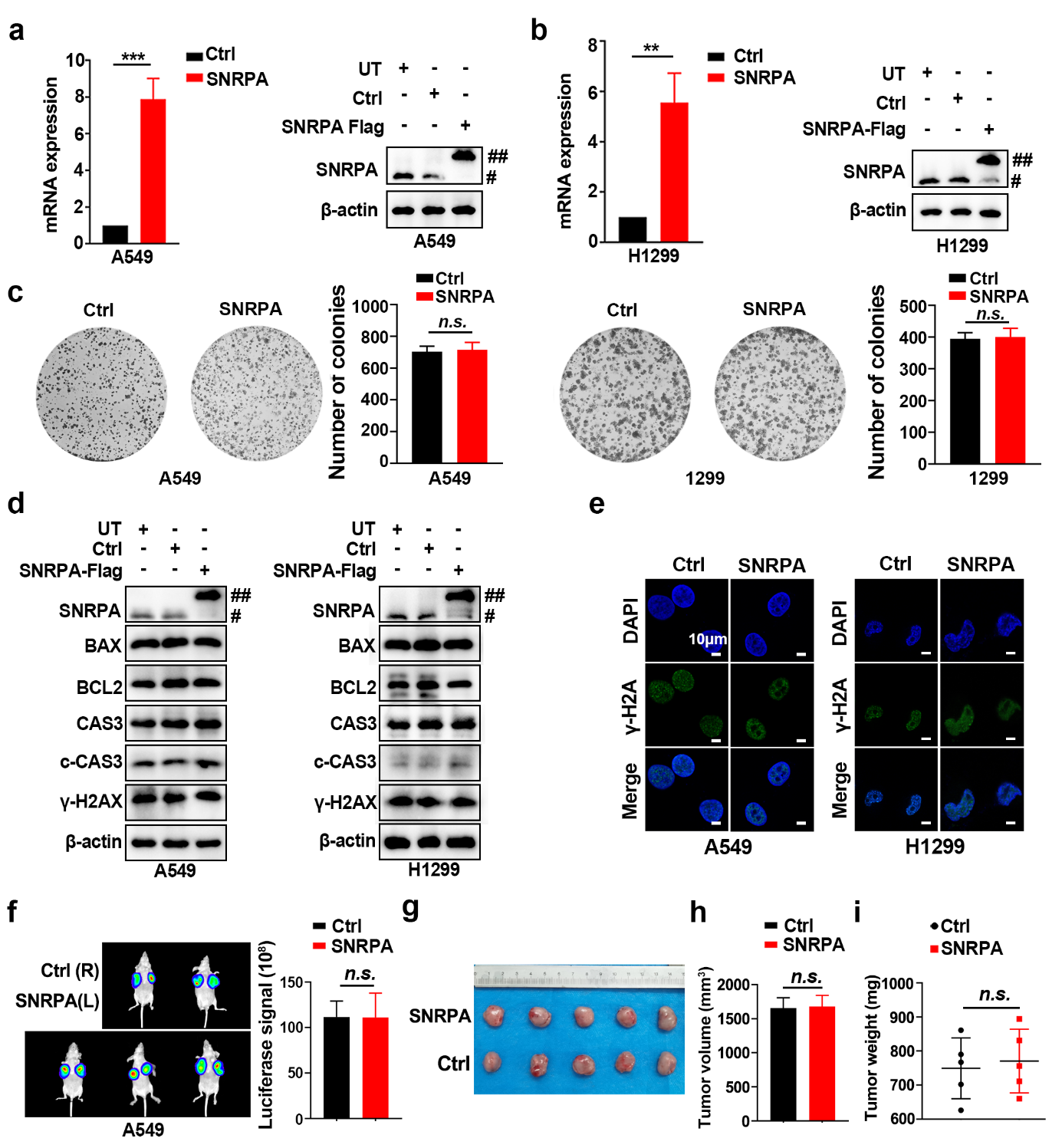


**Figure S4. Overexpression of SNRPA does not affect the proliferation, apoptosis and DNA damage of LUAD cells without cisplatin.** a, b) qRT-PCR and Western blot techniques were utilized to measure SNRPA expression in engineered clones, including overexpressing SNRPA clone (SNRPA) and a control vector clone (Ctrl), derived from A549 and H1299 cells. c) Plate colony formation assays were conducted to evaluate the proliferative capacity of Ctrl and SNRPA cells without cisplatin. d) The levels of apoptotic markers and γ-H2AX were investigated in LUAD cells with SNRPA overexpression via immunoblotting techniques. ## represents exogenous SNRPA—SNRPA-Flag; # represents endogenous SNRPA. e) Co-localization of γ-H2AX and DAPI immunofluorescence showed γ-H2AX nuclear foci. f) Bioluminescent imaging of xenograft tumors from negative control (Ctrl) or SNRPA-overexpressing (SNRPA) cells were captured at 24 days without cisplatin treatment, along with images of the harvested xenograft tumors. Statistical evaluation of the luciferase signal from the xenograft tumors is represented in bar graphs. g‒i) Statistical analysis were conducted to determine the average tumor volume and weight among the different groups. Data are presented as the mean±SD (n=3). The *P* values in panels (a), (b), (c), (f), (h) and (i) were calculated by Student’s t-test. ***P* < 0.01; ****P* < 0.001; *n.s*. indicates no significance.


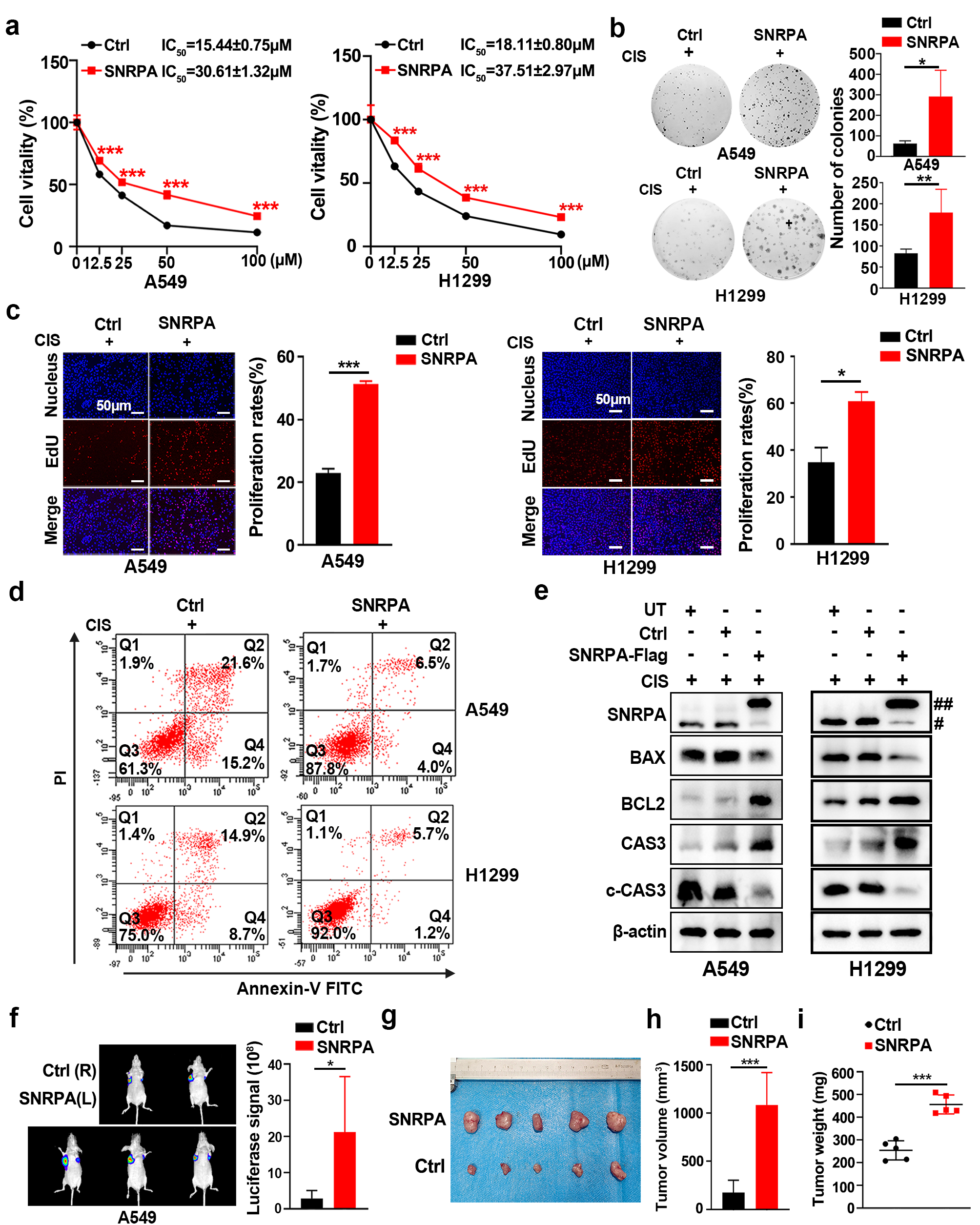


**Figure S5****. Overexpression of SNRPA induces cisplatin chemoresistance in cisplatin-sensitive LUAD cells.** a) The influence of SNRPA overexpression on the viability of LUAD cells treated with cisplatin was quantified using CCK-8 cell viability assays (n=4). b, c) Plate colony formation assays (b) and EdU assays (c) were conducted to evaluate the proliferative capacity of Ctrl and SNRPA derived from LUAD cells treated with cisplatin. d) Flow cytometry analysis was conducted to determine the extent of apoptosis induced by cisplatin in the indicated cells. e) The levels of apoptotic markers were investigated in LUAD cells with SNRPA overexpression by immunoblot. ## represents exogenous SNRPA—SNRPA-Flag; #represents endogenous SNRPA. f) Bioluminescence images of xenograft tumors from negative control (Ctrl) or SNRPA-overexpressing (SNRPA) cells were captured at 28 days following treatment, and images of the harvested xenograft tumors are shown. The statistical evaluation of the luciferase signal from the xenograft tumors is presented in the bar graphs. g‒i) Statistical analyses were conducted to determine the average tumor volume (h) and weight (i) among the different groups. All data are presented as the mean ± SD (n ≥ 3). The *P* values in panels (a) were calculated using two-way ANOVA. The *P* values in panels (b), (c), (f), (h) and (i) were calculated by Student’s t-test.**P* < 0.05; ***P* < 0.01; ****P* < 0.001.


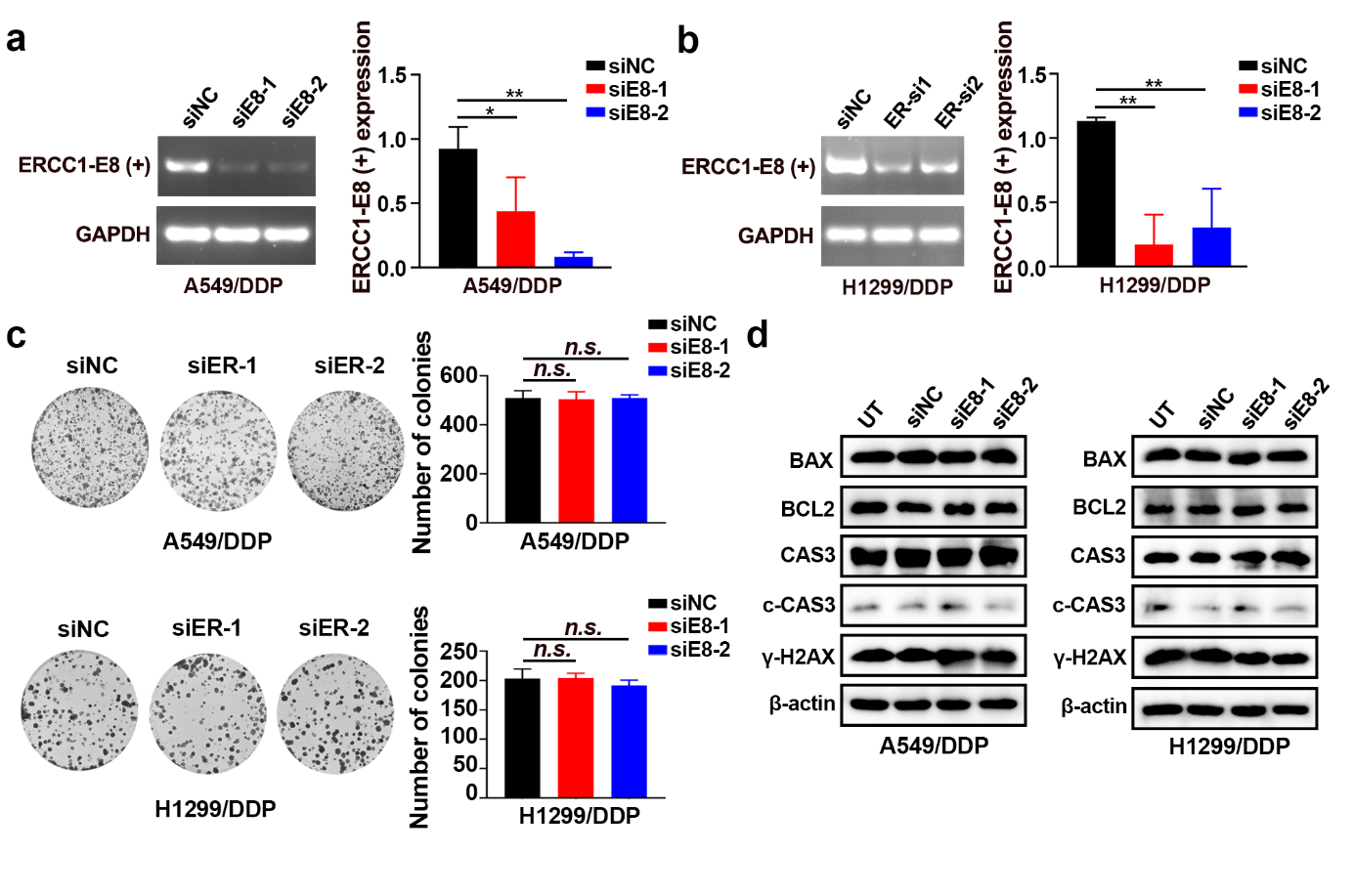


**Figure S6. Knockdown of** **ERCC1-E8 (+) could not influence the proliferation and apoptosis of cisplatin-resistant cells without cisplatin.** a, b) Agarose gel electrophoresis was employed to depict the expression levels of the ERCC1-E8 (+) transcript in the cisplatin-resistant cells after ERCC1-E8 (+) knockdown. Speciﬁc primers were designed against exon 7, 8 and 9 to examine the expression of ERCC1 transcripts containing exon 8 [named ERCC1-E8 (+) or E8 (+)]. Cropped blots are shown for the indicated ERCC1 isoforms or GAPDH. For uncropped blots, see Supporting Information. c) The colony-forming ability of specified cell populations without cisplatin was assessed. Colonies were visualized using crystal violet staining (left panel). Quantitative analysis of colony counts is presented in the bar graphs (right panel). d) Western blot showed the expression of BAX, BCL2, CAS3, C-CAS3, which are known apoptosis-associated proteins, and γ-H2AX. All the data are presented as the mean± SD (n = 3). The *P* values in panels (a), (b) and (c) were calculated using one-way ANOVA. **P* < 0.05; ***P* < 0.01; *n.s.* indicates no significance.


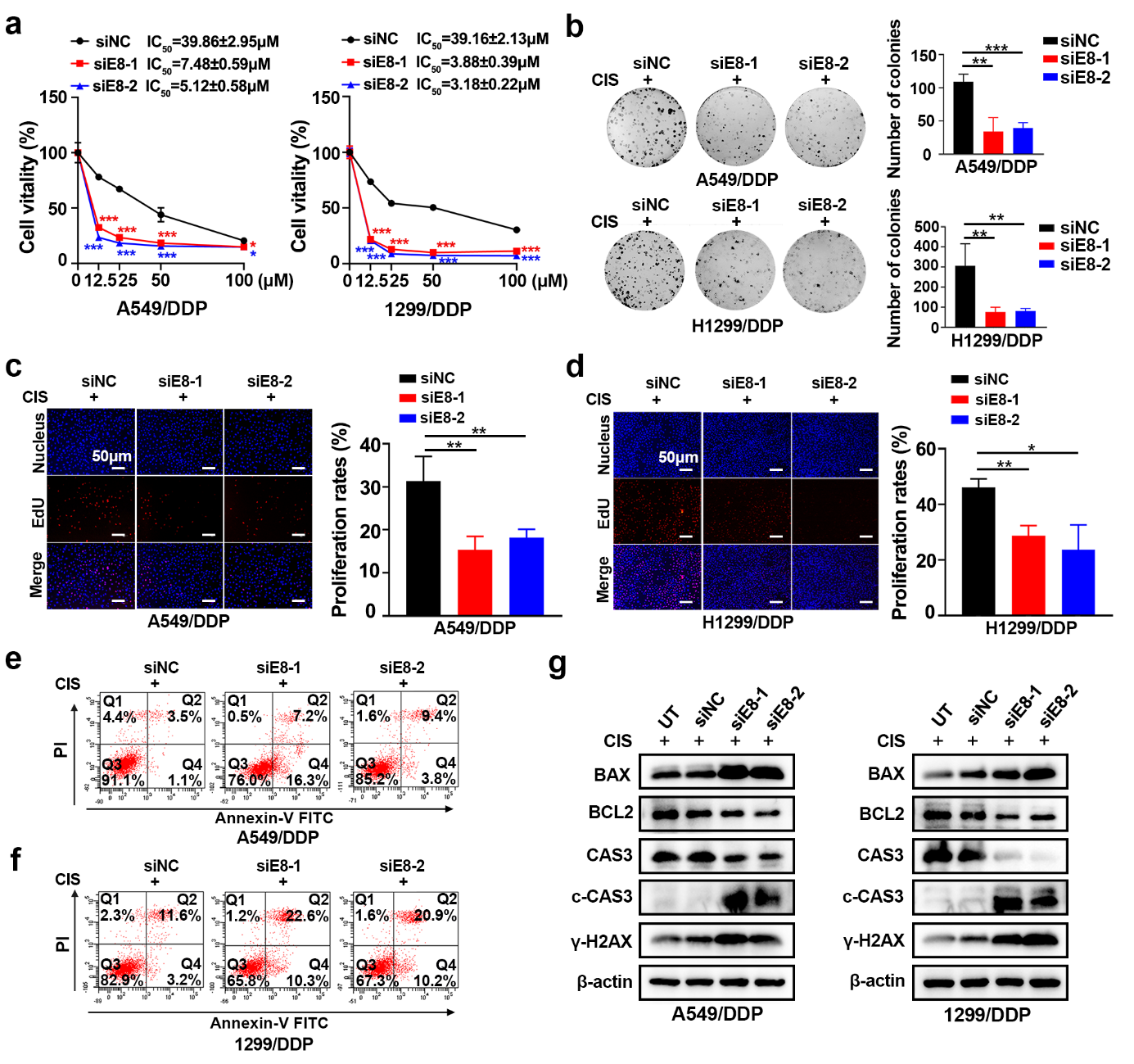


**Figure S7. Knockdown of ERCC1-E8 (+) reverses the resistance of cisplatin-resistant cells to cisplatin.** a) Cell viability was determined by performing a CCK-8 analysis after 48 hours of cisplatin treatment (n=4). b) The specified cells were subjected to a 14-day treatment with 10 µM cisplatin and analyzed using the colony formation test. Crystal violet was used to stain the colonies (left panel). The number of colonies was analyzed and was presented in bar graphs (right panel). c, d) EdU assays were conducted to assess the proliferation of the indicated cells in a 10 µM cisplatin solution. e, f) Representative images illustrating the Annexin V-FITC/PI staining of the specified cells following a 24-hour treatment with 10 µM cisplatin. g) The expression of apoptosis-associated proteins and γ-H2AX was determined. All data are presented as the mean ± SD (n ≥ 3). The *P* values in panels (b), (c) and (d) were calculated using one-way ANOVA. The *P* values in panels (a) were calculated using two-way ANOVA. **P* < 0.05; ***P* < 0.01; ****P* < 0.001.


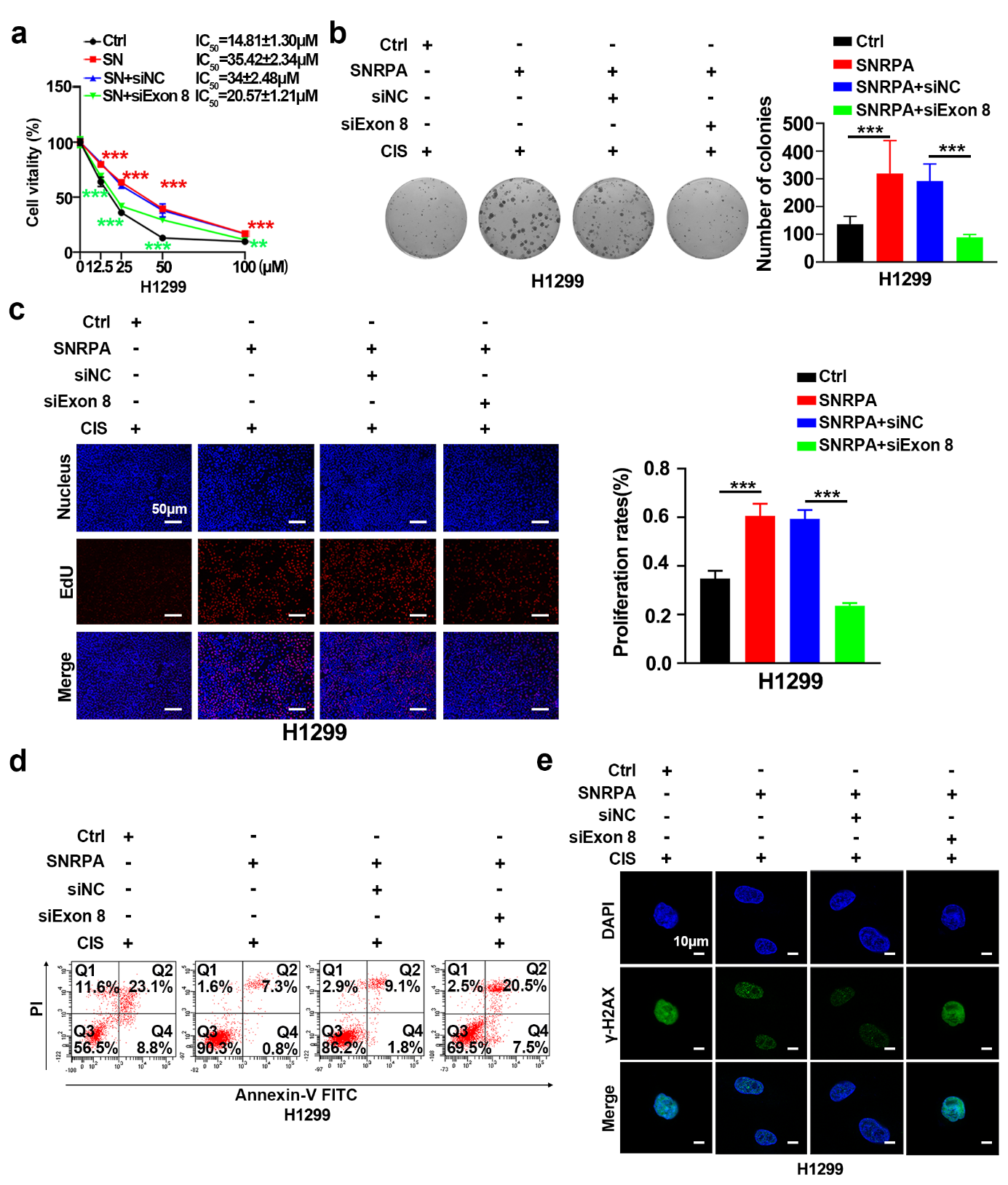


**Figure S8.** **Silencing of ERCC1-E8 (+) significantly reversed SNRPA-enhanced cisplatin resistance in 1299 cells.** On the basis of overexpression of SNRPA, ERCC1-E8 (+) expression in cisplatin-resistant cells was ERCC1-E8 (+) knockdown using H1299 cells. a) Cell viability in the designated cells was quantified utilizing a CCK-8 following a 48-hour exposure to cisplatin in 1299 originated cells (n=4). b) The indicated cells were treated with 10 µM cisplatin for 14 days, according to the colony formation test. Crystal violet was used to dye the colonies (left panel). The statistical analysis of the number of colonies is displayed in the bar graphs (right panel). c) EdU assays showed the proliferation of indicated cells in a 10 µM cisplatin solution. d) Representative images (left panel) depicted Annexin V-FITC/PI staining of the specified cells subjected to a 24-hour treatment with 10 µM cisplatin. e) Immunofluorescence co-localization of γ-H2AX and DAPI. All data are presented as the mean ± SD (n ≥ 3). The *P* values in panels (b) and (c) were calculated using one-way ANOVA. The *P* values in panels (a) were calculated using two-way ANOVA. ****P* < 0.001.


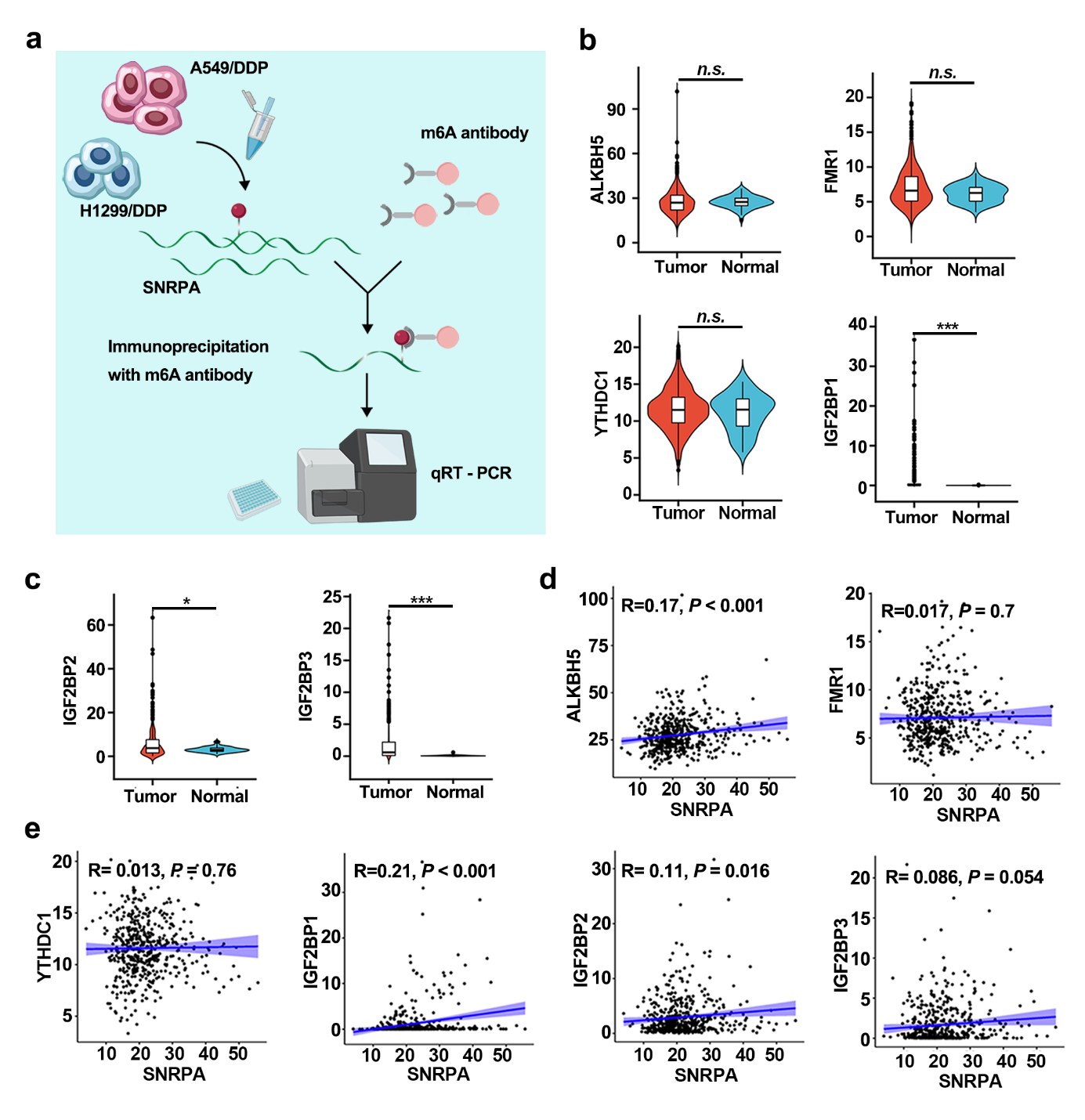


**Figure S9. ELAVL1 is selected as the best candidate for m^6^A regulators of SNRPA.** a) Schematic diagram of the MeRIP assay. b, c) The violin plots illustrated the expression levels of, the exception of ELAVL1, other 6 genes expression in tumor and normal tissues retrieved from the TCGA-LUAD database. d, e) Pearson correlation analysis of, the exception of ELAVL1, other 6 genes expression and SNRPA expression in TCGA-LUAD dataset. All data are presented as the mean ± SD (n > 3). The *P* values in panels (b) and (c) were calculated by Student’s t-test.**P* < 0.05; ****P* < 0.001; *n.s.* indicates no significance.

**
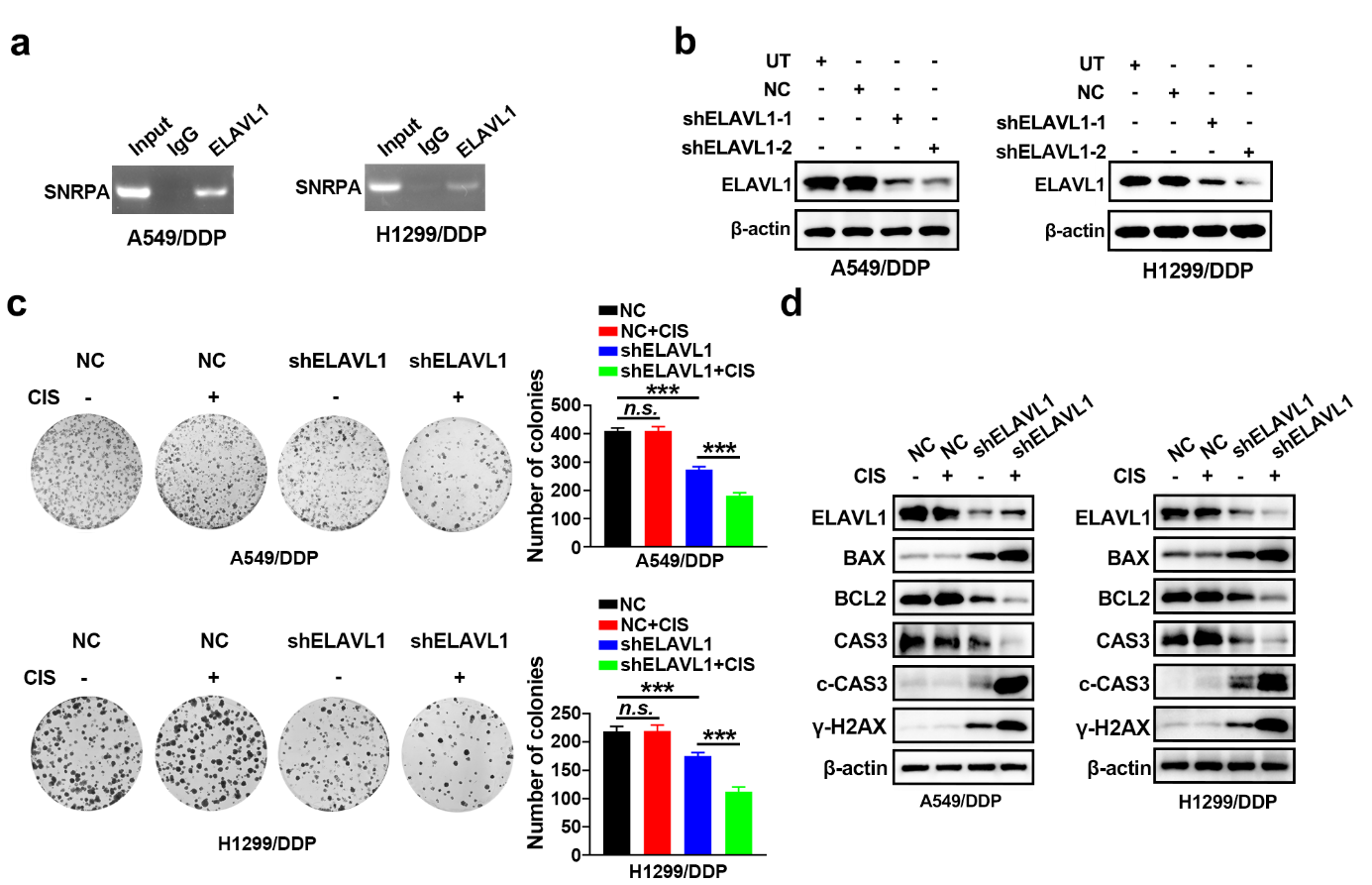
**

**Figure S10. Knockdown of ELAVL1 synergizes with cisplatin to enhance cisplatin sensitivity in LUAD cells.**  a) Agarose gel electrophoresis was employed to depict the expression levels of SNRPA obtained via RIP. Cropped blots are shown for the indicated SNRPA. For uncropped blots, see Supporting Information. b) The expression levels of ELAVL1 were evaluated in A549/DDP and H1299/DDP cells that were transfected with NC, shELAVL1-1, or shELAVL1-2 using Western blot. c) The colony-forming ability of specified cell populations with/without cisplatin were assessed. Crystal violet staining facilitated the visualization of colonies (left panel). Quantitative analysis of colony counts is presented in the bar graphs (right panel). d) Western blot performed the expression of apoptosis-associated proteins and γ-H2AX. Data are presented as the mean ± SD (n=3). The *P* values in panels (c) were calculated using one-way ANOVA. ****P* < 0.001; *n.s.* indicates no significance.

**
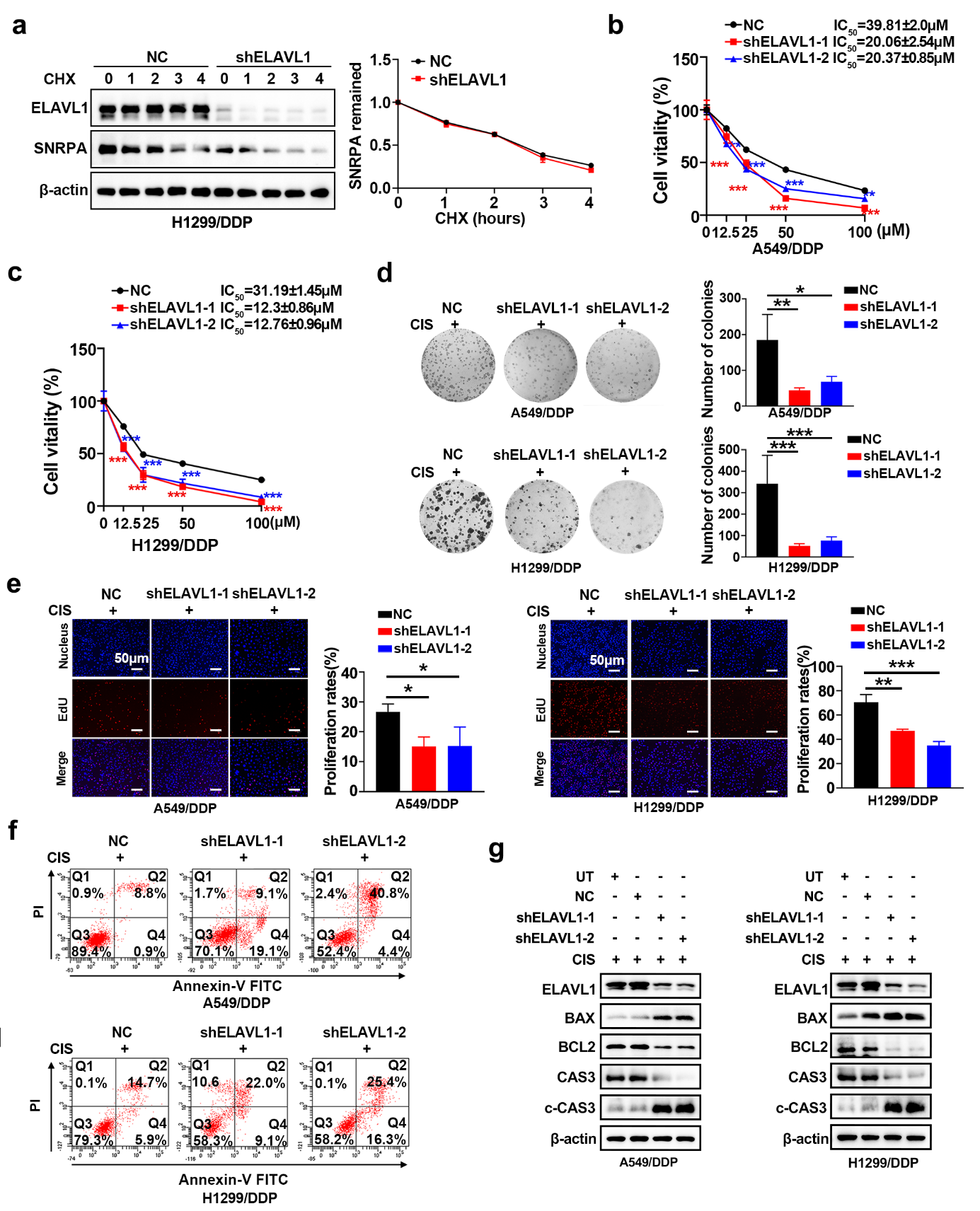
**

**Figure S11. ELAVL1 depletion reduced furtherance of cisplatin chemoresistance in LUAD cells.**  a) The stability of the SNRPA protein displayed by Western blot in 1299/DDP-derived cells treated with CHX in different time gradients. b, c) The viability of LUAD cells in the presence of cisplatin was evaluated using CCK-8 assays to assess the impact of ELAVL1 knockdown (n=4). d) The cells under investigation were treated with 10 µM cisplatin for 14 days, the effects of which were quantified using a colony formation assay. Staining of resultant colonies were stained with crystal violet (left panel). The right panel features bar graphs depicting the statistical evaluation of the number of colonies. e) EdU assays showed the proliferation of the indicated cells in a 10 µM cisplatin solution. f) Representative micrographs illustrating Annexin V-FITC/PI staining of the cells of interest following a 24-hour incubation with 10 µM cisplatin. g) Expression profiles of proteins associated with apoptotic pathways. All data are presented as the mean ± SD (n ≥ 3). The *P* values in panels (d) and (e) were calculated using one-way ANOVA. The *P* values in panels (b) and (c) were calculated using two-way ANOVA. **P* < 0.05; ***P* < 0.01; and ****P* < 0.001.


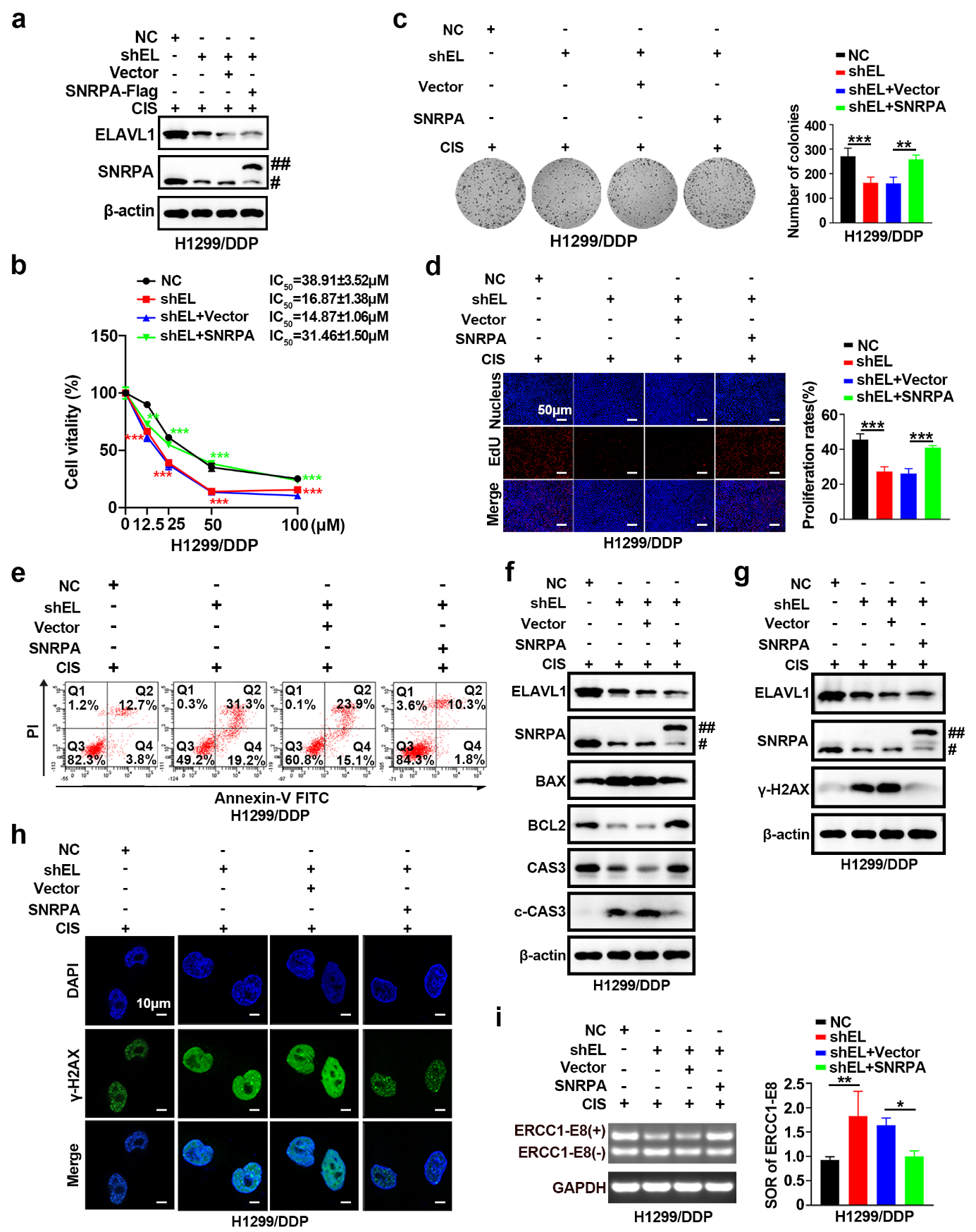


**Figure S12.** **ELAVL1 promotes cisplatin resistance, DNA repair and ERCC1-E8 (+) expression in 1299/DDP cells in an SNRPA-dependent manner.** a) Western blot analysis was utilized to determine the levels of ELAVL1 and SNRPA expression in cisplatin-resistant cell lines following the overexpression of SNRPA based on the knockdown of ELAVL1. b) The viability of LUAD cells in the presence of cisplatin was evaluated using CCK-8 assays (n=4). c) The delineated cells were exposed to 10 µM cisplatin for 14 days as part of the colony formation assay. The cell colonies were stained with crystal violet (left panel), and the quantification of the colonies was statistically analyzed; the results are presented in the form of bar graphs (right panel). d) EdU assays showing the proliferation of the indicated cells in a 10 µM cisplatin solution. e) Representative images (left panel) depicted Annexin V-FITC/PI staining of the specified cells subjected to 24 hours of treatment with 10 µM cisplatin. The right panel displays the statistical analysis in bar graphs. f, g) The expression of apoptosis-associated proteins and γ-H2AX were examined. h) Immunofluorescence co-localization of γ-H2AX and DAPI. (i) Agarose gel showing the SOR of ERCC1-E8. Cropped blots are shown for the indicated ERCC1 isoforms or GAPDH. For uncropped blots, see Supporting Information. All data are presented as the mean ± SD (n ≥ 3). The *P* values in panels (b) were calculated using two-way ANOVA. The *P* values in panels (c), (d) and (i) were calculated using one-way ANOVA. **P* < 0.05; ***P* < 0.01; and ****P* < 0.001.


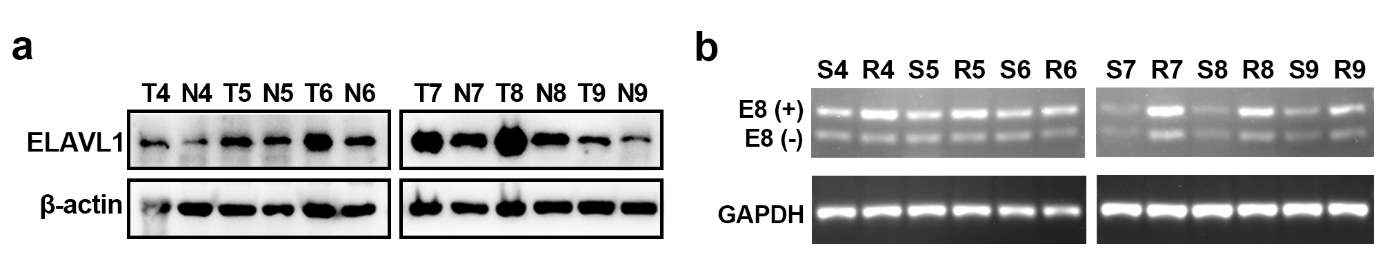


**Figure S13.** **Expression of ELAVL1 and ERCC1-E8 (+)/(-) in human tissues.** a) Immunoblotting analysis revealing the protein expression of ELAVL1 using fresh frozen LUAD (T) and normal adjacent (N) tissue samples. b) Agarose gel electrophoresis showing the expression of ERCC1-E8 (+) and ERCC1-E8 (-) in fresh frozen cisplatin-sensitive (S) (n = 9) and cisplatin-resistant (R) (n = 9) tissue samples. Cropped blots are shown for the indicated ERCC1 isoforms or GAPDH. For uncropped blots, see Supporting Information.
